# Hydrogen-Rich Water Ameliorates Metabolic Disorder via Modifying Gut Microbiota in Impaired Fasting Glucose Patients: A Randomized Controlled Study

**DOI:** 10.3390/antiox12061245

**Published:** 2023-06-09

**Authors:** Bing Liang, Le Shi, Dongyue Du, Hua Li, Ning Yi, Yue Xi, Jianjiao Cui, Ping Li, Hongbin Kang, Mami Noda, Xuejun Sun, Jiankang Liu, Shucun Qin, Jiangang Long

**Affiliations:** 1 Center for Mitochondrial Biology and Medicine, The Key Laboratory of Biomedical Information Engineering of Ministry of Education, School of Life Science and Technology, Xi’an Jiaotong University, Xi’an 710049, China; 2 The First Affiliated Hospital of Jinzhou Medical University, Jinzhou 121001, China; 3 State Key Laboratory of Virology, Wuhan Institute of Virology, Chinese Academy of Sciences, Wuhan 430071, China; 4 Department of Surgical Nursing, School of Nursing, Jinzhou Medical University, Jinzhou 121001, China; 5 The Third Affiliated Hospital of Jinzhou Medical University, Jinzhou 121001, China; 6 Laboratory of Pathophysiology, Graduate School of Pharmaceutical Sciences, Kyushu University, Fukuoka 812-8582, Japan; 7 Department of Naval Medicine, Naval Medical University, Shanghai 200433, China; 8 School of Health and Life Sciences, University of Health and Rehabilitation Sciences, Qingdao 266071, China; 9 Taishan Institute for Hydrogen Biomedicine, Shandong First Medical University and Shandong Academy of Medical Sciences, Taian 271000, China

**Keywords:** impaired fasting glucose, hydrogen-rich water, metabolomics, gut microbiota, correlation analysis

## Abstract

Objective: Molecular hydrogen (H_2_) exhibits antioxidant, anti-inflammatory and anti-apoptotic effects, and has shown benefits in glucose and lipid metabolism in certain animal metabolic disorder models. However, the potential benefits of H_2_ treatment in individuals with impaired fasting glucose (IFG) has seldom been studied. This randomized controlled study (RCT) aims to investigate the effects of hydrogen-rich water (HRW) on IFG subjects and explore the underlying mechanism involved. Methods: Seventy-three patients with IFG were enrolled in a randomized, double-blind, placebo-controlled clinical study. These patients were assigned to receive either 1000 mL per day of HRW or placebo pure water (no H_2_ infusion) for a duration of eight weeks. Metabolic parameters and fecal gut microbiota were assessed at baseline (week 0) and at week 8. A combined analysis of metabolomics and intestinal microbiota was conducted to investigate the correlation between the effect of H_2_ on the metabolisms and the diversity of intestinal flora in the IGF patients. Results: Both pure water and HRW demonstrated a significant reduction in fasting blood glucose in IFG patients, with a significant difference between pure water and HRW after eight weeks. Among IFG patients with abnormal pre-experimental fatty liver, 62.5% (10/16) in the HRW group and 31.6% (6/19) in the pure water group achieved remission. Furthermore, 16S RNA analysis revealed HRW-modified gut microbiota dysbiosis in the fecal samples of IGF patients. Through Pearson correlation analysis, the differential gut microbiota obtained by 16S analysis was found to be highly correlated with nine metabolites. Conclusion: H_2_ slightly improved metabolic abnormalities and gut microbiota dysbiosis, providing a novel target and theoretical basis for the prevention and treatment of blood glucose regulation in patients with IFG.

## 1. Introduction

The ninth edition of the Global Diabetes Survey, published by the International Diabetes Federation (IDF), reveals a concerning trend of increasing diabetes cases worldwide. In 2019, the survey found that approximately 463 million adults, aged 20–79, were affected by diabetes. The age-standardized prevalence of diabetes stood at 8.3%, and it is projected to reach 9.2% and 9.6% by 2030 and 2045, respectively. Moreover, an estimated 373.9 million individuals within the same age range were found to have impaired fasting glucose (IFG) in 2019. This number is predicted to rise to 453.8 million and 548.4 million in 2030 and 2045, respectively. Notably, nearly half (48.1%) of the IFG population was under the age of 50, with a significant portion (28.3%) falling within the 20–39-year-old age bracket. Among the countries with the highest number of individuals affected by IFG, China ranked first with 54.5 million cases, followed by the United States with 37.4 million and Indonesia with 29.1 million [1]. Recent epidemiological research indicates that China has a diabetes prevalence of 11.2%. Furthermore, the study reveals that 9.7% of individuals between the ages of 24 and 64 in China have prediabetes, as determined by impaired glucose tolerance and impaired fasting blood glucose levels [2].

In recent years, basic and clinical research on hydrogen (H_2_) medicine has developed rapidly [3,4]. Oxidative stress is widely recognized as a significant pathophysiological factor in diabetic complications [5], making antioxidant therapy a promising avenue for diabetic complications [6,7]. Hyperglycemia-induced mitochondrial electron leak is a key source of endogenous reactive oxygen species (ROS), which can lead to vascular endothelial cell damage and the activation of multiple glucose metabolism pathways [8]. It has been observed that glucose overload can induce cell damage through oxidative stress, and the resulting oxidative stress is implicated in the pathogenesis of various diabetic complications [5]. While an appropriate level of ROS can promote insulin secretion, excessive ROS may trigger a strong oxidative stress response, downregulate insulin gene expression, and directly damage β cells, ultimately leading to the development of diabetes and its associated complications [9].

To date, most clinical studies on H_2_ therapy for diabetes have primarily focused on basic biochemical parameters. In animal models, the administration of hydrogen-rich water (HRW) has demonstrated beneficial effects such as reducing body weight, liver oxidative stress, fatty liver, blood glucose, insulin, and triglyceride levels. These effects are attributed to the enhanced expression of fibroblast growth factor 21 (FGF21), a hepatic hormone that promotes fatty acid and glucose expenditure [10]. In clinical trials, HRW showed significant reductions in modified low-density lipoprotein (LDL) cholesterol by modifying the net negative charge of LDL, very low-density lipoprotein (VLDL) in blood, and 8-isoprostaglandin (8-isoPG) in urine. HRW has also been associated with decreased levels of oxidized LDL and free fatty acid (FFA), as well as increased plasma adiponectin (ADPN) and extracellular superoxide dismutase (EC-SOD) levels [11]. Furthermore, HRW administration has shown potential in reducing liver fat accumulation in patients with non-alcoholic fatty liver disease compared to placebo administration [12]. However, the relevant metabolic pathways and mechanisms of H_2_ have not been thoroughly explored. In recent years, the application and advancement of metabolomics in diabetes research have provided a comprehensive and systematic understanding of the pathogenesis of diabetes.

It has been found that H_2_ produced by gut flora shortens colonic transit, particularly in the proximal colon rather than the distal colon [13]. Numerous studies have also indicated the association between gut microbiota-mediated H_2_ production and gastrointestinal diseases as well as metabolic disorders. In addition, H_2_ administration in vitro had significant effects on the gut environment, including the short-chain fatty acid (SCFA) content and microbial composition. For instance, Higashimura et al. found that mice treated with H_2_-dissolved alkaline-electrolyzed water (AEW) exhibited an increased abundance of *Butyricimonas virrosa strain* MT12, as well as elevated concentration of SCFAs isobutyric acid and propionic acid. This suggests a potential association between the increased presence of these SCFAs and the *Butyricimonas virrosa* strains. Furthermore, *Candidatus arthromitus* was also significantly increased in AEW-treated mice, which plays a key role in the maturation of the gut’s innate and adaptive immune systems [14]. Presumably, under conditions with an impaired production of H_2_ due to intestinal damage, exogenous H_2_ via HRW could modify gut microbiota and ameliorate metabolism. Further investigation is warranted to explore this hypothesis in the future.

This study was designed as a randomized, double-blind, placebo-controlled clinical trial. By collecting 73 IFG patients and giving 41 of them a pure water and 32 of them an HRW intervention, we investigated whether H_2_ could improve abnormal glucose metabolism. Further, from the perspective of intestinal flora, we explored the potential relationship between metabolic pathways and related clinical effects.

## 2. Methods and Materials

### 2.1. Screening and Grouping of Subjects

#### 2.1.1. Subjects

A total of 73 subjects with IFG participated in the study, with 32 subjects assigned to the hydrogen water treatment group and 41 subjects assigned to the pure water control group. The participants were recruited from the Third Affiliated Hospital of Jinzhou Medical University. The study was approved by the Hospital Ethics Committee, with the Ethics Committee Opinion No. JYDSY-KXYJ-IEC-2020-002.

#### 2.1.2. Inclusion Criteria

IFG diagnostic criteria were applied using the diagnostic criteria for impaired fasting blood sugar proposed by the WHO in 1999: a fasting blood sugar of 6.1 to 7.0 mmol/L.

#### 2.1.3. Exclusion Criteria

(1) Patients with type 1, type 2 diabetes and other special types of diabetes.
(2) Patients with obvious abnormal liver and kidney function or those who have recently used drugs that affect liver and kidney function.
(3) Patients during pregnancy and breastfeeding.
(4) Subjects with a blood relationship with other subjects.
(5) Not living in Jinzhou within 3 years before the start of the study.
(6) Patients who have used antioxidants such as vitamin E, vitamin C, folate tablets, etc., within 3 months prior to the start of the study.
(7) Patients who used antibiotics within 1 month of the start of the study.
(8) Patients in an acute stress state caused by other diseases (such as fractures, elevated blood sugar caused by trauma, etc.).
(9) Patients with a history of alcoholism, cancer, HIV infection, stroke, myocardial infarction and other serious chronic disease.

### 2.2. Study Design

The HRW used for the intervention was produced by Wenzhou Hydrogen Source Technology Co., Ltd (Wenzhou, China). Two production batches were used in this study. The production dates were 9 May 2020 and 28 October 2020. Placebo pure water was also supplied by the same company. The food safety supervision sampling inspection report label was DC20330300300932063. The hydrogen concentration was detected using a methylene blue dissolved hydrogen concentration measuring agent (Miz.Co, Fujisawa, Japan) [15]. Patients consumed HRW (hydrogen concentration: 1.4 mg/L) at 1 L/Day for 8 weeks. Randomized, double-blind and placebo (pure water, no H_2_ infusion) controls were taken [12]. The patients were tested for relevant clinical and laboratory test indices before and after participating in the trial. The following parameters were measured at baseline (week 0) and after 8 weeks.

#### 2.2.1. Basic Index Collection

The basic information survey on patients, health status survey such as chronic disease history, and physical examination information such as height and blood pressure were collected before and after 8 weeks. The basic blood test indices primarily included the total protein, albumin, globulin, total bilirubin, direct bilirubin, creatinine, uric acid, lactate dehydrogenase, creatine kinase, red blood cell count, hemoglobin, glycated hemoglobin, white blood cell count, platelets, etc. 

To ensure the compliance of the patients and the effect of the trial, the research group designed and administered a patient questionnaire application. At the beginning of the study, the subjects completed a questionnaire on basic information and dietary structure. Throughout the trial, their daily water intake and body weight were recorded using a mobile app. Patients’ facial photos were collected once a week [16], and the Pittsburgh Sleep Quality Index Questionnaire and Exercise Habits Questionnaire was completed once a week. The reliability and validity of the questionnaire was tested among 20 subjects prior to the experiment. This questionnaire had good internal reliability (Cronbach’s alpha = 0.783). The Kaiser–Meyer–Olkin measure of sampling adequacy (0.704) and Bartlett’s test of sphericity (*p* < 0.001) both showed that the questionnaire data were suitable for factor analysis. 

#### 2.2.2. Glucose Metabolism-Related Index Detection

The fasting blood glucose level was one of the indices measured in the study. A disposable vacuum blood collection tube was utilized to collect 2–3 mL of venous blood, which was promptly sent to the medical laboratory on the same morning as the collection. The collected specimens were centrifuged at a low speed for 3 min at 3000–5000 rpm. After centrifugation, the specimens were divided into two layers by an inert separating gel, and the upper layer was serum. If immediate detection was not feasible, the serum could be refrigerated at 4–8 °C for a maximum of 48 h before analysis. 

#### 2.2.3. Lipid Metabolism-Related Index Detection

The detection methods for the relevant biochemical indices were the same as those related to glucose metabolism, including total cholesterol, triglyceride, high-density lipoprotein, low-density lipoprotein and very low-density lipoprotein, etc. An abdominal liver ultrasound (DC-70, Minday, Shenzhen, China) was used for fatty liver detection. A visceral fat meter (HDS-2000, Omron, Kyoto, Japan) was used for visceral fat detection. The body weight, body mass index and abdominal circumference were manually measured at the medical examination center.

#### 2.2.4. Inflammatory Factor Detection

The levels of serum interleukin-1β (IL-1β), interleukin-2 (IL-2), interleukin-6 (IL-6), and TNF-α were detected by using enzyme-linked immunosorbent assay (ELISA) kits (FANKEL BIO CO., Ltd., Shanghai, China) according to the manufacturer’s protocols.

#### 2.2.5. Metabolism-Related Index Detection

Tandem mass spectrometry detection items were used for the detection of amino acids, fatty acids, and carnitine. An unsupervised model of Principal Component Analysis (PCA) and a supervised model of partial least squared discriminant analysis (PLS-DA) were used to evaluate the holistic metabolomic alterations and identify important variables for diabetes mellitus by using SIMCA-P v12.0 (Umetrics, Umea, Sweden). Furthermore, the importance in projection (VIP) values were calculated, which signified the contribution of variables towards discrepancies between the HRW and placebo groups. In this study, VIP values were used to screen the biomarkers. A permutation test with 200 iterations was used to validate the rationality of the PLS-DA model. The normality of all the parameters for patients and healthy individuals was checked via the Shapiro–Wilk test. A *t*-test was used to detect the differences between groups. In addition, the Mann–Whitney test was performed for nonparametric variables. The Benjamini–Hochberg-based false discovery rate (FDR) was used to adjust the *p* values towards multiple hypothesis testing [17]. Volcano plots were used to select the biomarkers. The parameters with VIP > 1, adjusted *p*-values < 0.05 and fold change (FC) >1.2 or FC < −1.2 between patients and healthy individuals were applied to select the important metabolites. Selected biomarkers were used to screen significant biomarkers for the pure water and HRW groups [18]. Statistical analysis was conducted using SAS software (Cary, NA, USA).

#### 2.2.6. Intestinal Flora Detection

For intestinal flora detection, according to the clinical glucose and fatty-acid-related indexes, we chose 8 participants whose glucose and fatty liver were significantly alleviated in HRW group, and 4 participants whose glucose and fatty liver were not improved in placebo group. A total of 24 fresh fecal samples were collected from 12 participants pre- and post-treatment in placebo and HRW groups using sterile fecal collectors. The samples were immediately placed on ice, the middle and inner parts of the sample were intercepted and sorted, and then stored at −80 °C until analysis. The feces samples were sent to Biotree Co., Ltd. (Shanghai, China) for gut microbiota analysis. In brief, 0.25–0.5 g feces were used for DNA extraction by using the TIANamp Bacterial Kit (Tiangen Biochemical Technology, Beijing, China) according to the manufacturer’s instructions [19]. The PCR method was used to amplify the bacterial 16S RNA V3-V4 region using the primers 338F 5′-ACTCCTACGGGAGGCAGCA-3′ and 806R 5′-GGACTACHVGGGTWTCTAAT-3′. As 1 participant in the placebo group and 2 participants in the HRW group dropped out, finally 3 participants were in the placebo group and 6 participants were in the HRW group, and in total 18 samples were used for further analysis. Illumina Miseq analysis was used to sequence the purified PCR products on an Illumina HiSeq platform by Hiseq. Raw fastq files were quality-filtered using QIIME1 (version 1.9.1). Operational Units (OTUs) were clustered with a 97% similarity cut-off using USEARCH (version 10.0). The phylogenetic affiliation of each 16S rRNA gene sequence was analyzed by a Bayesian classifier using Silva.132 as reference database with a confidence threshold of 70%.

### 2.3. Statistical Analysis

Statistical analysis of blood test index data was performed using the SPSS 26.0 (SPSS Inc., Chicago, IL, USA), and GraphPad Prism 9.0 (GraphPad Software., San Diego, CA, USA) was used for statistical chart drawing. Continuous variables that conform to the normal distribution were expressed as the mean ± standard error of the mean (mean ± SEM), while non-normal variables were expressed as the median of the interquartile range (median ± IR). The variables that satisfy the normal distribution were compared using unpaired-samples *t* test and paired-samples *t* test, respectively, according to the experimental design, and the variables that did not satisfy the normal distribution were compared using the Mann–Whitney test and the Wilcoxon signed rank sum test, respectively. *p* < 0.05 was used as the standard to judge whether the difference between groups was significant. O2PLS modeling was performed using SIMCA software (Umetrics, Sweden) to detect the association between microbiota and metabolites. In the O2PLS model, microbiota data were defined as the X matrix, metabolite data were defined as the Y matrix, and the association between microbiota data and metabolites was evaluated by mapping the data from the X matrix to the Y matrix. Pearson correlation was used to test the relationship between microbiota and metabolites, and the results were further visualized by R software (version 3.6.1) (Alphabet, Palo Alto, CA, USA) with the “corrplot” package.

## 3. Results

### 3.1. Screening of Subjects and Results of Questionnaire Survey

**Figure 1:**
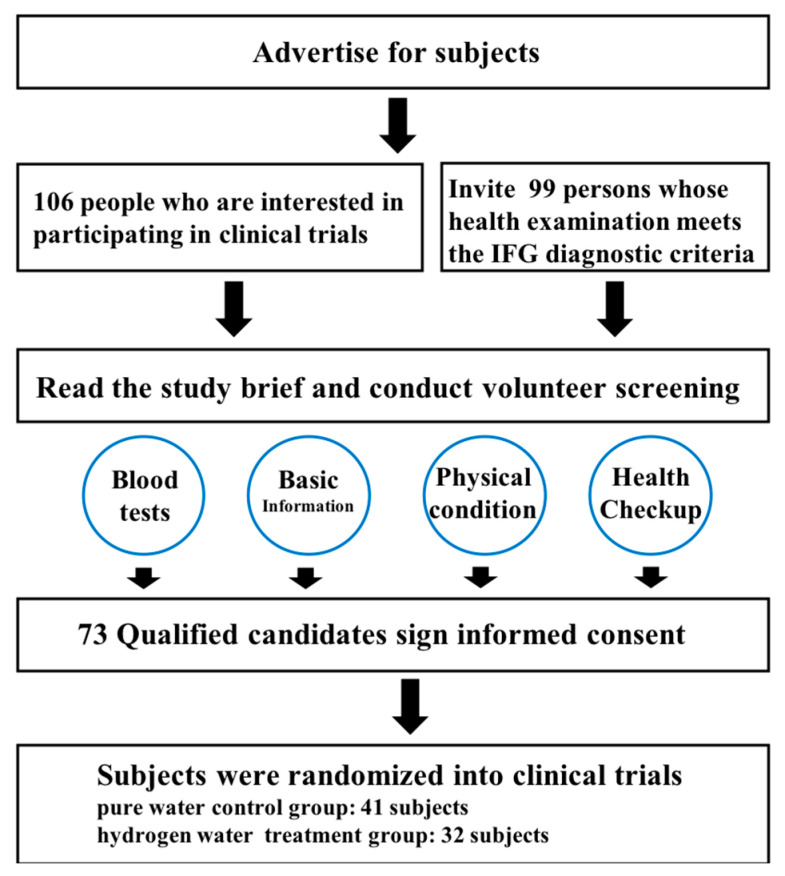
The flow chart for participant enrollment/follow-up.

A total of 106 subjects with IFG were enrolled, ranging in age from 35 to 55 years old, with approximately 50% males and females. After verification, seven subjects who did not meet the inclusion criteria were excluded. There were 26 subjects in the dropout group due to various factors and 73 subjects in the normal group, including 32 in the hydrogen water treatment group and 41 in the pure water control group (Figure 1). The baseline characteristics of the studied patients are listed in Table S1.

The H_2_ concentrations of 5 days, 1 month, 2 months, and 4 months after HRW production were detected, all of which reached the hydrogen concentration of the packaging label of 1.6 mg/mL. The hydrogen concentration met the experimental requirements of 1.4 mg/mL, while the placebo water was free of H_2_ (Figure S1). 

In our study, the majority of the subjects had a relatively regular diet, and generally had the habit of eating breakfast. Approximately 85.4% of the subjects reported regularly eating breakfast, while 12.6% occasionally ate breakfast, and only 1.9% rarely or never ate breakfast. The intake of fruits and vegetables was good, although the variety could be more abundant. The intake of nuts should be increased. Most of the participants consumed few snacks and cold drinks. A total of 76.7% of the subjects had a relatively regular diet, with little or no midnight snack, 63.1% of the subjects had few snacks and cold drinks in their diet, and no one ate snacks or drank cold drinks every day. The intake of pure water was high, and most people ensured that they drank enough water every day. The data could be obtained via https://jinshuju.net/f/yPQjDP/r/pIUqO7 (accessed on 1 June 2022).

The sleep quality of the subjects was generally good, and the need for sleep aids was minimal. Most subjects reported feeling awake and energetic without experiencing drowsiness. A total of 52.7% of the subjects thought their sleep quality was better, 29.9% thought their sleep quality was good, and 14.6% thought their sleep quality was poor. A total of 96.1% of subjects did not require drug hypnosis, over half of the respondents (55.4%) reported not feeling sleepy regularly in the past week, and 20.2% experienced feeling sleepy once or twice a week. Due to personal workloads and other reasons, most of the subjects had limited physical activity and an uncertain exercise schedule, often restricted to the evening. Most subjects had a low awareness of the importance of exercise and had minimal exercise demands. In this research, 45.8% of the subjects exercised occasionally, 24.9% exercised two to three times a week, 21.9% exercised more than four times a week, and the number of people who exercised once a week was the least at only 7.4%. Over half of the participants (51.3%) preferred to exercise at night, 32.9% were uncertainty about the timing, and only 5.6% chose to exercise in the afternoon. The data could be observed via https://jinshuju.net/f/b6YifU/r/XdEaSw (accessed on 1 June 2022).

### 3.2. Effects of HRW on Glucose Metabolism-Related Indicators

**Figure 2:**
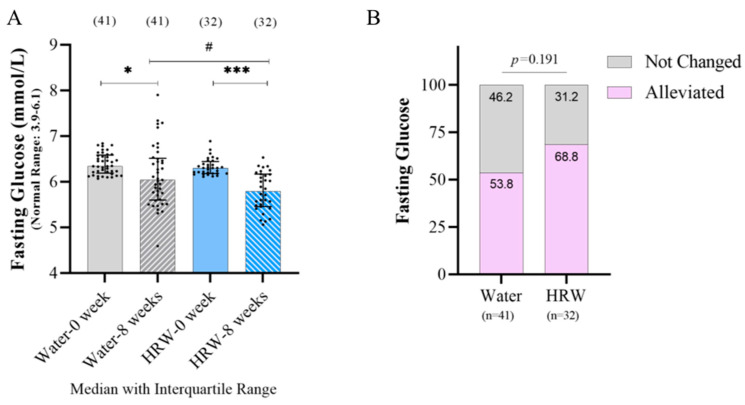
The effect of HRW on glucose metabolism-related indicators. (**A**) Fasting glucose changes between each group after eight weeks of treatment (Mann–Whitney test and Wilcoxon test). (**B**) Effective ratio of falling within the normal range after eight weeks of treatment (Pearson Chi-square). * Significantly different between weeks 0 and 8 in each group (*: *p* ≤ 0.05; ***: *p* ≤ 0.001). # Significantly different between HRW and placebo groups in the same week (#: *p* ≤ 0.05). Black dot represents sample value.

The subjects were chosen according to the inclusion criteria (fasting blood sugar 6.1 to 7.0 mmol/L), rather than their adherence to a normal distribution. Therefore, we first verified whether each indicator followed the normal distribution, and chose a more appropriate test according to the data attributes. The changes in body composition and biomarkers after eight weeks of treatment with HRW and the placebo are shown in Table 1. The fasting glucose level significantly decreased in the HRW group, though the placebo water group also showed a decline after eight weeks; however, the HRW group’s fasting glucose level decreased more obviously (Figure 2A). After eight weeks of treatment, the effective ratio of falling within the normal range (<6.1 mmol/L) is shown in Figure 2B. A total of 68.8% (22/32) in the HRW group and 53.8% (22/41) in the placebo group returned to normal glucose levels after eight weeks, but there was no significant difference between HRW and placebo groups.

### 3.3. Effects of HRW on Lipid Metabolism-Related Indicators

**Figure 3:**
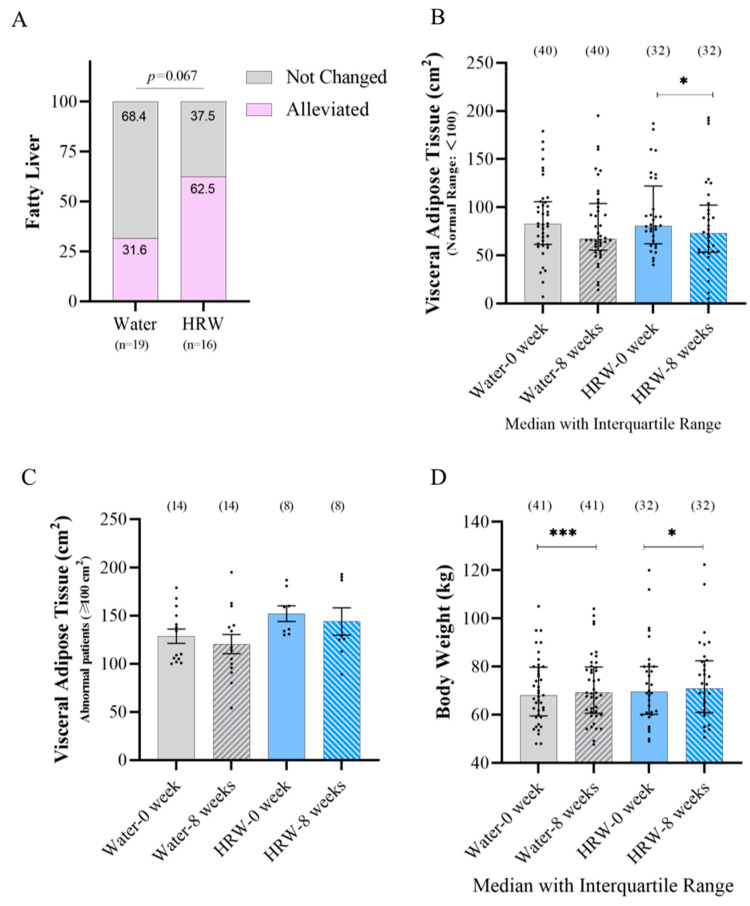
The effect of HRW on lipid metabolism-related indicators. (**A**) After eight weeks of treatment, the effective ratio of alleviating symptoms in patients with abnormal fatty liver at the beginning of the study (Pearson Chi-square). (**B**) Visceral adipose tissue changes in all patients after eight weeks of treatment (Mann–Whitney test and Wilcoxon test). (**C**) After eight weeks of treatment, the changes in patients with abnormal visceral adipose tissue at the beginning (paired and unpaired *t*-test). (**D**) Body weight changes after eight weeks of treatment (Mann–Whitney test and Wilcoxon test). * Significantly different from between weeks 0 and 8 in each group (*: *p* ≤ 0.05; ***: *p* ≤ 0.001). Black dot represents sample value.

Nineteen subjects in the placebo group and sixteen subjects in the HRW group had fatty liver at the beginning of the study. After eight weeks of treatment, a total of 62.5% (10/16) in the HRW group and 31.6% (6/19) in the placebo group were alleviated, and a marginally significant difference was identified (Figure 3A). The visceral adipose tissue of all the subjects was significantly reduced after eight weeks of HRW treatment, but when separately compared with the abnormal patients (visceral adipose tissue: 100 cm^2^ at the beginning of the study), there were no significant differences among the groups (Figure 3B,C). Body weight was significantly increased after eight weeks of treatment, but such an increase appeared to be more obvious in the placebo group rather than in the HRW group (Figure 3D). 

### 3.4. Effects of HRW on the Gut Microbiota

**Figure 4:**
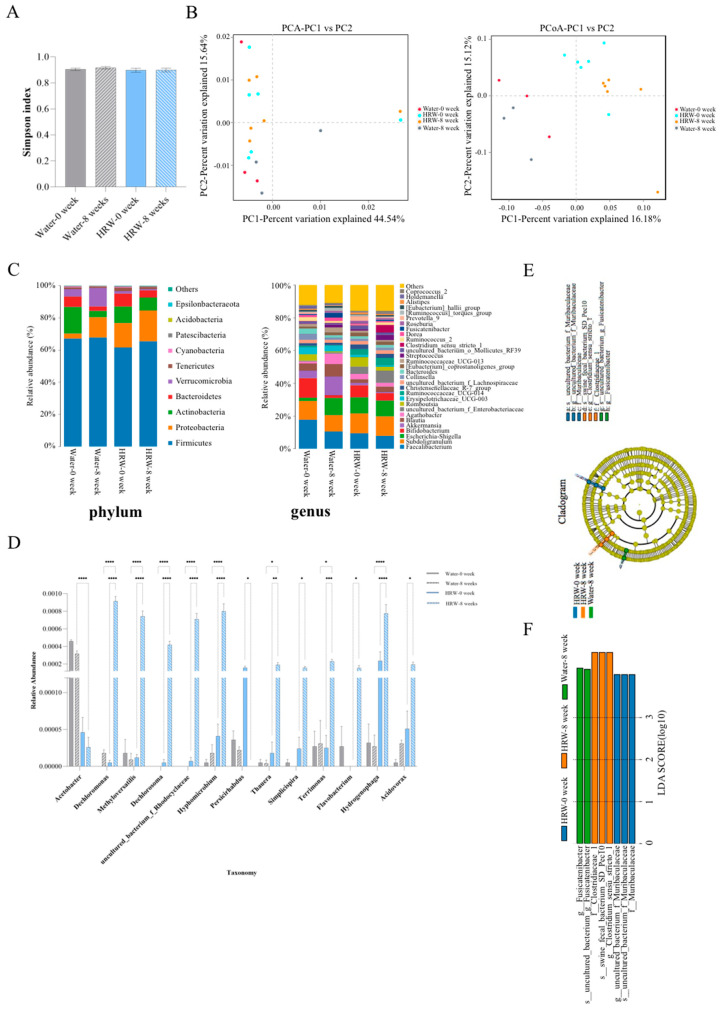
The effect of HRW on the gut microbiota after eight weeks of treatment. (**A**) Simpson α-diversity index analysis. (**B**) PCA and PCoA scores. (**C**) The microbial composition and abundance analysis at the phylum and genus levels. (**D**) The effect of HRW on intestinal flora. (**E**) LEfSe analysis of the main flora in each group. Each ring represents the next lower taxonomic level (phylum to genus). (**F**) Results of the LEfSe analysis (LDA > 10). Data are presented as mean ± SD, and evaluated by using one-way ANOVA followed by Tukey’s post hoc test (*: *p* ≤ 0.05; **: *p* ≤ 0.01; ***: *p* ≤ 0.001, ****: *p* ≤ 0.0001).

A total of 1,439,422 pairs of reads were obtained from the sequencing of 18 samples, and a total of 1,432,945 clean reads were produced after paired-end read quality control and splicing. Each sample produced at least 79,275 clean reads, with an average of 79,608 clean reads. The α-diversity analysis showed that there was no significant change in the abundance of bacterial flora before and after placebo treatment in either group (Figure 4A). PCA analysis showed that the samples in each group were not well separated; thus, the samples were further analyzed by the PCoA method, and there was an obvious separation between the samples in each group (Figure 4B).

At the phylum level, *Firmicutes*, *Proteobacteria*, *Actinobacteria*, *Bacteroidetes*, *Verrucomicrobia*, *Tenericutes*, *Cyanobacteria*, *Patescibacteria*, *Acidobacteria*, and *Epsilonbacteraeota* were highly abundant. At the genus level, the microbial diversity in the HRW group was increased compared with the placebo group, but there was no significant difference between the two groups. In addition, *Faecalibacterium*, *Subdoligranulum*, *Escherichia_Shigella*, *Bifidobacterium*, *Akkermansia*, *Blautia*, *Agathobacter*, *uncultured_bacterium_f_Enterobacteriaceae*, *Romboutsia*, and *Erysipelotrichaceae_UCG-003* were highly abundant bacteria (Figure 4C).

Through the ANOVA analysis, at the genus level, we screened the differential flora of the HRW group before and after treatment as well as the difference between the placebo group and the HRW group after treatment. Compared with the placebo group, HRW significantly increased the relative abundance of the genera *Dechloromonas*, *Methyloversatilis*, *Dechlorosoma*, *Rhodocyclaceae*, *Hyphomicrobium*, *Thauera*, *Simplicispira*, *Terrimonas*, *Flavobacterium*, *Hydrogenophaga* and *Acidovorax*, while significantly decreasing the relative abundance of the genera *Acetobacter* and *Persicirhabdus* (Figure 4D). The LEfSe analysis uncovered the predominant microbial profile in the placebo and HRW groups. The HRW group included three main bacterial groups at the family, genus and species levels, namely, *Clostridiaceae*, *swine_fecal_bacterium_SD_Pec10*, and *Clostridium_sensu_stricto*. (Figure 4E,F).

### 3.5. Correlation Analysis between Gut Microbiota and Metabolites

**Figure 5:**
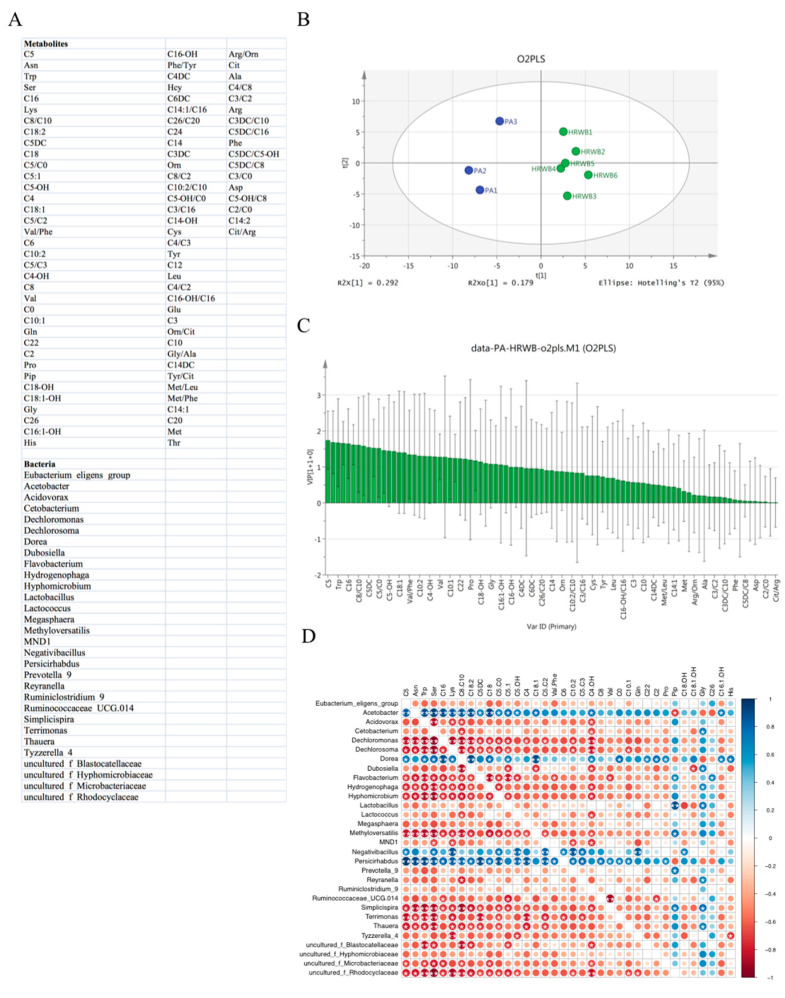
Correlation analysis between gut microbiota and metabolites based on the O2PLS model. (**A**) The metabolites and intestinal flora categories; (**B**) O2PLS model score; (**C**) VIP value of metabolites; (**D**) Pearson correlation analysis of gut flora and metabolites. Strong correlations are indicated by large circles, whereas weak correlations are indicated by small circles. The color of the scale bar represents the nature of the correlation, with blue indicating a positive correlation and red indicating a negative correlation (white star means significant of correlation, *: *p* ≤ 0.05; **: *p* ≤ 0.01).

An O2PLS model was constructed to analyze the association between differential metabolites and gut microbes in the placebo water and hydrogen water groups. As no significantly different metabolites were detected using tandem mass spectrometry (Table S2), we chose all the detected metabolites between the placebo water and hydrogen water groups for the O2PLS model. The gut microbes in the O2PLS model were the different microbiota between the two groups at the genus level. The metabolites and intestinal flora categories are shown in Figure 5A. The R2 and Q2 values of the model were 0.502 and 0.244, respectively, revealing that the O2PLS model could be used for metabolite-gut microbiota analysis and prediction (Figure 5B). The VIP values of the metabolites were ranging from 0.011 to 1.744. Among them, 36 metabolites had VIP values > 1.0, highlighting C5, Asn, Trp, Ser, C16, Lys, C8/C10, C18:2, C5DC, C18, C5/C0, C5-OH, C4, C18:1, C5/C2, Val/Phe, C6, and C10:2 as important downstream metabolites influenced by changes in intestinal flora due to HRW (Figure 5C). To further explore the interaction between intestinal flora and these metabolites (VIP > 1.0), Pearson correlation analysis was conducted. It revealed a high correlation between the gut microbes obtained by 16S analysis, including *Acetobacter*, *Dechloromonas*, *Dechlorosoma*, *Flavobbacterium*, *Hydrogenophaga*, *Hyphomicrobium*, *Methyloversatilis*, *Persicirhabdus*, *Simplicispira*, *Terrimonas*, *Thauera*, and *uncultured_ bacterium_f_Rhodocyclaceae,* and nine metabolites, C5, Asn, Trp, Ser, Lys, C8/C10, C18:2, C5/C0, and C4-OH were highly correlated (Figure 5D).

## 4. Discussion

In this study, we demonstrated that HRW intake for eight weeks reduced serum fasting glucose levels, and there was a significant difference between the HRW group and the placebo group, suggesting that H_2_ has a potential effect on blood glucose regulation in patients with IFG.

However, unlike further clinical studies [11,20], our results show that even the placebo water treatment also led to a reduction in fasting glucose after eight weeks, possibly for the following reasons: (1) Subjects were all recruited through the physical examination of healthy people. Many factors can cause glucose level fluctuations at the IFG stage [21]. Subjects may pay more attention to their own health status after knowing they have IFG, leading to improvements in blood glucose levels. (2) After the subjects were enrolled to participate in the clinical trial, to ensure their compliance, we designed a questionnaire online filling system, with the relevant information and dietary structure questionnaire. To a certain extent, it may have interfered with the subjects’ diet, sleep, exercise and other daily habits and styles, promoting healthier lifestyle choices that could have positively impacted blood glucose levels [5]. (3) The questionnaire results indicated that the subjects generally had good sleep quality, which could contribute to better blood glucose control. In addition, studies have identified that the adequate intake of water has a preventive effect on diabetes, with copeptin being a potential mechanism. The water intake stimulates the secretion of copeptin, leads to increased ACTH and cortisol secretion, enhances glycogenolysis and gluconeogenesis, and regulates insulin and glucagon secretion. All of these factors contribute to the prevention and treatment of metabolic diseases, including diabetes [22,23,24]. In our study, subjects consumed an additional 1000 mL of water daily, ensuring sufficient hydration, and potentially enhancing glucose metabolism and reducing blood glucose levels. These factors collectively contribute to the observed reduction in fasting glucose levels, even in the placebo group, highlighting the importance of lifestyle modifications and hydration in blood glucose regulation.

Regarding the influence of HRW on lipid metabolism, we found that HRW alleviated fatty liver in comparison with the placebo group. Interestingly, a total of 31.6% (6/19) of the placebo group were alleviated. Additionally, HRW could reduce the visceral adipose tissue of all the subjects after eight weeks, but as for the abnormal visceral adipose tissue subjects, the trend was not significant. This may due to a limited number of abnormal subjects or a need for a longer treatment period with HRW to observe significant changes. Nevertheless, our findings provide evidence that HRW plays an important role in reducing fatty liver. This suggests its potential as a therapeutic intervention for improving lipid metabolism and liver health. Further research with large sample sizes and longer treatment durations may provide more conclusive results regarding the effect of HRW on visceral adipose tissue in individuals with abnormal levels.

In a four-week clinical trial, there were no significant differences observed in body weight, BMI, and body circumference changes between treatment groups. Compared with the placebo group, H_2_ could respectively reduce body fat percentage and arm fat indices, indicating that hydrogen reduced body fat, but did not reduce body weight [25]. However, in the present study, both the HRW group and placebo group showed a significant increase in body weight. However, the increase in the placebo group was more pronounced. This can be attributed to the timing and duration of the trial, which took place from September to February in the northern region of China. During this seasonal transition, there were significant temperature changes that could affect the energy demand of the human body. The appetite of the human body is generally improved in winter compared with summer, the basal metabolic rate of the human body is lower, and energy is more likely to accumulate in the body as fat; so, subjects appeared to gain weight. It is important to consider these factors when interpreting the results of the study and understanding the impact of HRW on body weight.

Other research has reported that HRW can reduce serum LDL and ApoB levels, while improving dyslipidemia-impaired high-density lipoprotein cholesterol (HDL) function and reducing oxidative stress, and may have beneficial effects on preventing underlying metabolic syndrome [12]. However, in the current trial, the change in ApoB was not obvious. Subjects with abnormal total cholesterol (TC) at the beginning showed a downward trend after HRW treatment, but there was no significant difference due to the small number of patients or short time of HRW treatment. There was no significant change in HDL and LDL in this trial. The results indicated that the role of hydrogen in the treatment of obesity requires further discussion.

There is growing evidence that human microbiota, especially the gastrointestinal microbiota, have a potential role in the etiology and pathological outcome of type 2 diabetes and its complications [26]. In our trial, α- and β-diversity analyses revealed differences in microbial composition and structure within each group. Through the ANOVA and LEfSe analysis, at the genus level, the differential flora of the HRW group before and after HRW, as well as the placebo group after placebo, were screened. Furthermore, numerous studies have demonstrated that gut microbiota regulate aging and aging-related diseases, such as diabetes, obesity, Alzheimer’s disease, and tumors by manipulating the metabolism in intestine and other tissues [27,28,29]. Although we did not detect different metabolites between the placebo and HRW groups by using tandem mass spectrometry, we observed that Glu, Cit and C14:2 appeared to be the predominant metabolites in the HRW group (Tables S3 and S4). These findings suggest that Glu, Cit and C14:2 may be the key molecules involved in the protective effect of HRW on IFG patients, though these metabolites were insignificant after Bonferroni correction. In our study, we found that *Methyloversatills*, which has been identified in human neck squamous cell carcinoma patients [30], was significantly increased in IFG patients treated with HRW and negatively correlated with several metabolites, such as C5, Asn, Trp, Ser and C8/C10. *Flavobacterium*, which has been reported to be reduced in NAFLD patients [31] and HFD animal models [32], was elevated in IFG patients after HRW supplementation, and negatively correlated with Trp, C18 and C5:1. *Dechloromonas*, which was shown to decline with age during the first six months of life in infants [33], increased in IFG patients treated with HRW and negatively correlated with some metabolites, such as C5, Asn, Trp, Ser, Lys, C8/C10, C182, and C4-OH. All these results indicate that *Methyloversatills, Flavobacterium* and *Dechloromonas* might be involved in the metabolism of HRW-treated IFG patients. Although the genera identified in IFG patients, *Thauera*, *Terrimonas*, *Simplicispira*, *Hyphomicrobium*, *Hydrogenophagea*, and *Dechlorosoma* are commonly found in the environment and have not yet been reported in diabetes, obesity or other aging-related diseases, our results highlight their potential role in understanding HRW-IFG patients’ effects. In addition, the modified microbiota could change the level of biomarkers they produced, including H_2_, which is an antioxidant [34], indicting a positive-feedback regulation mechanism of H_2_ and gut microbiota.

This study is designed as a randomized controlled clinical trial to evaluate the effectiveness of H_2_ intervention in patients with IFG. The treatment group was the HRW group, and the pure water group was the placebo group. The blood glucose value of the hydrogen water group could decrease by 0.8, set bilateral α = 0.05, and the certainty was 90%. Sample size was calculated according to the formula (n = 2(z_α_ + z_β_)^2^ ∗ σ^2^/δ^2^) for estimating the sample content of measurement data in the experimental study. n = 27 cases were obtained. Considering the 1:1 randomized grouping, that is, 27 cases were required for each of HRW group and placebo group, and considering the 15% deactivation group, at least 32 cases were required for each of the HRW and placebo groups, and a total of at least 64 subjects were included, suggesting that the number of cases in our study was enough. Nevertheless, this sample size calculation is only an estimate, and this research is still limited because only a small number of IGF cases was included. In this trial, we did not specifically select patients with gastrointestinal symptoms, and we could not confirm the fully defined effect of HRW. However, it has been reported that H_2_ is thought to modulate the gut microbiota and contribute to intestinal normalization [35]. Notably, the participants for intestinal flora detection were much smaller; this may negatively impact the reliability of the gut microbiota results. In terms of the sleep questionnaire, a total of 106 subjects participated, seven subjects who did not meet the inclusion criteria were excluded, and 713 pieces of data were obtained by analyzing the questionnaire survey from 99 subjects. Most of the subjects reported better or good sleep quality, with 52.7% considering their sleep quality as better and 29.9% considering it as good. Only 14.6% of subjects reported poor sleep quality. Compared to the placebo group, the HRW group reported a greater decrease in the time to fall asleep, as well as an increase in sleep duration and self-reported good sleep quality, suggesting that HRW may improve sleep. In general, the effects of HRW on glucose metabolism are very small and the number of cases is low. This raised the uncertainty of the biological and physiological relevance of our findings. This warrants further confirmation to avoid potentially artificial positive results.

## 5. Conclusions

In conclusion, our study suggests that H_2_ showed potentially beneficial effects on glucose metabolism, possibly by interfering with the gut microbiota of patients with IFG. However, it is important to note that the effect of H_2_ may be smaller compared to the impact of lifestyle intervention such as diet, sleep, and exercise. To validate our findings, further long-term and well-designed clinical studies are needed. Additionally, the underlying mechanisms of H_2_ effects in treating metabolic syndrome remain to be further investigated. It would be valuable to conduct animal studies involving microbiota transplantation to confirm the role of gut microbiota and metabolites in mediating the effects of H_2_.

## Figures and Tables

**Table 1:** Changes in body composition and body biomarkers between the HRW and placebo group after eight weeks of treatment.

	Hydrogen-Rich Water Group		Placebo Water Group		Values Presented
	(n = 32)		(n = 41)		
	0 Week	8 Weeks	0 Week	8 Weeks	
Weight (kg)	69.50 ± 19.78	70.85 ± 21.53 *	68.10 ± 20.30	69.20 ± 19.30 ***	Median ± IR
Body mass index (kg/m^2^)	25.26 ± 4.29	25.09 ± 4.15	24.66 ± 3.41	24.63 ± 3.36	Mean ± SD
Abdominal girth (cm)	85.37 ± 12.59	84.47 ± 12.65	83.90 ± 10.94	84.22 ± 11.32	Mean ± SD
Visceral Adipose Tissue (cm^2^)	80.50 ± 60.00	73.00 ± 49.00 *	82.50 ± 44.50	66.00 ± 49.00	Median ± IR
Fasting glucose (mmol/L)	6.30 ± 0.26	5.79 ± 0.71 *** #	6.34 ± 0.40	6.04 ± 0.92 *	Median ± IR
Creatinine (μmol/L)	67.96 ± 18.19	69.51 ± 18.47	69.22 ± 10.64	69.49 ± 11.91	Mean ± SD
Uric acid (μmol/L)	354.43 ± 134.35	356.06 ± 117.36	381.02 ± 126.73	355.96 ± 109.44 **	Mean ± SD
Total protein (g/L)	73.13 ± 4.43	73.12 ± 3.95	71.77 ± 4.19	72.42 ± 3.73	Mean ± SD
Globulin (g/L)	27.54 ± 3.66	27.04 ± 3.26	26.20 ± 3.11	26.18 ± 3.29	Mean ± SD
Albumin (g/L)	45.59 ± 2.36	46.07 ± 2.34	45.56 ± 2.52	46.23 ± 2.08	Mean ± SD
AST (IU/L)	20.50 ± 7.75	19.00 ± 7.25	20.00 ± 8.50	21.00 ± 6.50	Median ± IR
ALT (IU/L)	24.50 ± 17.50	20.00 ± 19.75	25.00 ± 19.50	23.00 ± 20.00	Median ± IR
Total bilirubin (μmol/L)	11.20 ± 7.75	11.60 ± 8.40	11.10 ± 8.15	11.70 ± 6.20	Median ± IR
Direct bilirubin (μmol/L)	2.55 ± 1.28	2.75 ± 1.20	2.40 ± 1.40	2.60 ± 1.55	Median ± IR
TC (mmol/L)	5.26 ± 0.69	5.11 ± 0.73	5.14 ± 0.96	5.22 ± 0.96	Mean ± SD
HDL (mmol/L)	1.25 ± 0.30	1.24 ± 0.32	1.24 ± 0.25	1.22 ± 0.27	Mean ± SD
LDL (mmol/L)	3.13 ± 0.63	3.13 ± 0.61	3.04 ± 0.73	3.11 ± 0.74	Mean ± SD
Triglycerides (mmol/L)	1.30 ± 1.05	1.14 ± 1.00	1.19 ± 0.80	1.42 ± 1.05	Median ± IR
GHb (%)	5.41 ± 0.56	5.32 ± 0.53	5.51 ± 0.51	5.36 ± 0.49 *	Mean ± SD
Insulin (U/mL)	12.57 ± 9.85	10.48 ± 8.25	10.45 ± 7.91	10.39 ± 9.55	Median ± IR
ALP (IU/L)	63.91 ± 16.07	65.56 ± 17.26	71.78 ± 17.09	73.59 ± 18.04	Mean ± SD
γ-GT (IU/L)	29.00 ± 21.00	26.00 ± 27.50	27.00 ± 28.00	25.00 ± 25.00 *	Median ± IR
LDH (U/L)	158.00 ± 46.75	144.00 ± 36.25 ***	161.00 ± 38.50	152.00 ± 30.50 *	Median ± IR
Creatine kinase (U/L)	82.00 ± 55.75	86.00 ± 52.00	99.00 ± 49.50	91.00 ± 56.00	Median ± IR
ApoA1 (mg/dL)	113.10 ± 26.95	117.90 ± 24.33 *	117.20 ± 25.00	115.60 ± 26.30 *	Median ± IR
ApoB (mg/dL)	96.33 ± 19.86	98.13 ± 19.14	94.51 ± 23.60	99.07 ± 23.53 *	Mean ± SD
Lipoprotein a (mg/L)	137.80 ± 270.85	137.95 ± 230.95	90.30 ± 205.35	138.60 ± 221.75	Median ± IR
RBC (10^9^/L)	4.63 ± 0.52	4.70 ± 0.62	4.69 ± 0.44	4.68 ± 0.47	Mean ± SD
Hb (g/L)	138.03 ± 14.91	141.81 ± 17.25	139.24 ± 16.72	140.10 ± 17.07	Mean ± SD
WBC (10^9^/L)	5.40 ± 1.55	5.15 ± 1.55	5.70 ± 1.70	5.30 ± 1.45	Median ± IR
Platelet (10^9^/L)	243.06 ± 53.51	232.38 ± 50.87 *	227.71 ± 43.20	226.29 ± 45.56	Mean ± SD
IL-1β (pg/mL)	26.38 ± 12.68	20.71 ± 6.24 ***	22.53 ± 12.34	18.86 ± 10.71 ***	Median ± IR
IL-2 (pg/mL)	31.97 ± 11.33	37.70 ± 13.59 ***	30.53 ± 6.98	38.37 ± 10.78 ***	Median ± IR
IL-6 (pg/mL)	171.76 ± 34.17	151.43 ± 27.06 ***	176.28 ± 25.30	150.07 ± 25.68 ***	Mean ± SD
GSH-Px (U/mL)	61.44 ± 25.05 ###	66.00 ± 26.95 * ###	48.18 ± 19.17	54.89 ± 17.03 ***	Median ± IR
TNF-α (pg/mL)	212.05 ± 46.81	161.67 ± 37.64 ***	208.95 ± 36.10	162.34 ± 34.72 ***	Mean ± SD
TAC (U/mL)	7.17 ± 3.23 #	8.02 ± 2.49 ***	5.70 ± 2.29	7.93 ± 3.49 ***	Median ± IR
MDA (nmol/mL)	9.00 ± 4.00	6.68 ± 3.34 ***	10.70 ± 4.96	6.64 ± 2.53 ***	Median ± IR
LPO (nmol/L)	2.61 ± 1.15	1.62 ± 1.30 ***	2.25 ± 0.98	1.94 ± 1.11 *	Median ± IR
C-RP (mg/dL)	0.27 ± 0.20	0.25 ± 0.15	0.25 ± 0.16	0.24 ± 0.15	Mean ± SD
Adiponectin (μg/mL)	7.64 ± 3.06	8.36 ± 2.05 *	6.96 ± 1.94	9.11 ± 1.99 ***	Mean ± SD
VLDL (ug/dL)	0.44 ± 0.10	0.52 ± 0.12 *	0.45 ± 0.12	0.51 ± 0.12 *	Mean ± SD
EC-SOD (U/mL)	19.78 ± 3.11	19.40 ± 4.13	19.86 ± 3.30	17.75 ± 2.70 **	Mean ± SD

Mean ± SEM for continuous variables with a normal distribution; Median ± IR for non-normal continuous variables. * Significantly different from week 0 of own group (*: *p* ≤ 0.05; **: *p* ≤ 0.01; ***: *p* ≤ 0.001). # Significantly different from the same week of different group (#: *p* ≤ 0.05; ###: *p* ≤ 0.001). AST: aspartate aminotransferase; ALT: alanine aminotransferase; TC: total cholesterol; HDL: high-density lipoprotein cholesterol; LDL: low-density lipoprotein cholesterol; GHb: glycated hemoglobin; ALP: alkaline phosphatase; γ-GT: gamma-glutamyl transpeptidase; LDH: lactate dehydrogenase; RBC: red blood cell count; Hb: hemoglobin; WBC: white blood cell count; GSH-Px: glutathione peroxidase; TAC: antioxidant capacity; MDA: malondialdehyde; LPO: lipid hydroperoxide; C-RP: C-reactive protein; VLDL: very low density lipoprotein; EC-SOD: extracellular-superoxide dismutase.

## Data Availability

The data presented in this study are available on reasonable request from the corresponding author.

## Supplementary Materials

The following supporting information can be downloaded at: https://www.mdpi.com/article/10.3390/antiox12061245/s1, Figure S1: The hydrogen concentration (mg/L); Table S1: The baseline characteristics of the studied patients with IGF; Table S2: The differential parameters between the HRW group and the placebo group after eight weeks of treatment; Table S3: The differential parameters between pre- and post-treatment in the placebo group; Table S4: The differential parameters between pre- and post-treatment in the HRW group; Table S5: The Pearson correlation analysis of gut flora and metabolites.

## References

[1] Saeedi P., Petersohn I., Salpea P., Malanda B., Karuranga S., Unwin N., Colagiuri S., Guariguata L., Motala A.A., Ogurtsova K. (2019). Global and regional diabetes prevalence estimates for 2019 and projections for 2030 and 2045: Results from the International Diabetes Federation Diabetes Atlas, 9th edition. Diabetes Res. Clin. Pract..

[2] Chinese Diabetes Socitey (2021). Guideline for the prevention and treatment of 2 diabetes mellitus in China (2020 edition). Chin. J. Diabetes Mellit..

[3] Zhang J.J., Liu C., Zhou L., Qu K.C., Wang R.T., Tai M.H., Wei J.C., Qi L.L., Wu F., Wang Z.X. (2012). A review of hydrogen as a new medical therapy. Hepatogastroenterology.

[4] Wang S.T., Bao C., He Y., Tian X., Yang Y., Zhang T., Xu K.F. (2020). Hydrogen gas (XEN) inhalation ameliorates airway inflammation in asthma and COPD patients. QJM Int. J. Med..

[5] Ceriello A., Testa R. (2009). Antioxidant anti-inflammatory treatment in type 2 diabetes. Diabetes Care.

[6] Tamasawa A., Mochizuki K., Hariya N., Saito M., Ishida H., Doguchi S., Yanagiya S., Osonoi T. (2015). Hydrogen gas production is associated with reduced interleukin-1β mRNA in peripheral blood after a single dose of acarbose in Japanese type 2 diabetic patients. Eur. J. Pharmacol..

[7] Iida A., Nosaka N., Yumoto T., Knaup E., Naito H., Nishiyama C., Yamakawa Y., Tsukahara K., Terado M., Sato K. (2016). The clinical application of hydrogen as a medical treatment. Acta Med. Okayama.

[8] Corkey B.E. (2012). Banting lecture 2011: Hyperinsulinemia: Cause or consequence?. Diabetes.

[9] Ley S.H., Hamdy O., Mohan V., Hu F.B. (2014). Prevention and management of type 2 diabetes: Dietary components and nutritional strategies. Lancet.

[10] Kamimura N., Nishimaki K., Ohsawa I., Ohta S. (2011). Molecular hydrogen improves obesity and diabetes by inducing hepatic FGF21 and stimulating energy metabolism in db/db mice. Obesity.

[11] Kajiyama S., Hasegawa G., Asano M., Hosoda H., Fukui M., Nakamura N., Kitawaki J., Imai S., Nakano K., Ohta M. (2008). Supplementation of hydrogen-rich water improves lipid and glucose metabolism in patients with type 2 diabetes or impaired glucose tolerance. Nutr. Res..

[12] Song G., Li M., Sang H., Zhang L., Li X., Yao S., Yu Y., Zong C., Xue Y., Qin S. (2013). Hydrogen-rich water decreases serum LDL-cholesterol levels and improves HDL function in patients with potential metabolic syndrome. J. Lipid Res..

[13] Jahng J., Jung I.S., Choi E.J., Conklin J.L., Park H. (2012). The effects of methane and hydrogen gases produced by enteric bacteria on ileal motility and colonic transit time. Neurogastroenterol. Motil..

[14] Higashimura Y., Baba Y., Inoue R., Takagi T., Uchiyama K., Mizushima K., Hirai Y., Ushiroda C., Tanaka Y., Naito Y. (2018). Effects of molecular hydrogen-dissolved alkaline electrolyzed water on intestinal environment in mice. Med. Gas Res..

[15] Seo T., Kurokawa R., Sato B. (2012). A convenient method for determining the concentration of hydrogen in water: Use of methylene blue with colloidal platinum. Med. Gas Res..

[16] Chilicka K., Rogowska A.M., Szygula R. (2021). Effects of topical hydrogen purification on skin parameters and acne vulgaris in adult women. Healthcare.

[17] Benjamini Y., Hochberg Y. (1995). Controlling the false discovery rate: A practical and powerful approach to multiple testing. J. R. Stat. Soc. B.

[18] Bai Q., Peng B., Wu X., Cao Y., Sun X., Hong M., Na R., Liu B., Li Q., Li Z. (2018). Metabolomic study for essential hypertension patients based on dried blood spot mass spectrometry approach. IUBMB Life.

[19] Hong X., Qin P.F., Yin J.C., Shi Y., Xuan Y., Chen Z.Q., Zhou X., Yu H., Peng D.H., Wang B. (2021). Clinical manifestations of polycystic ovary syndrome and associations with the vaginal microbiome: A cross-sectional based exploratory study. Front. Endocrinol..

[20] LeBaron T.W., Singh R.B., Fatima G., Kartikey K., Sharma J.P., Ostojic S.M., Gvozdjakova A., Kura B., Noda M., Mojto V. (2020). The effects of 24-week, high-concentration hydrogen-rich water on body composition, blood lipid profiles and inflammation biomarkers in men and women with metabolic syndrome: A randomized controlled trial. Diabetes Metab. Syndr. Obes..

[21] Zeng X., Liu H.F., Xiao M.F., Liu B., Liao J., Zhang J.N. (2019). Analysis of prevalence of thyroid cancer in patients with type 2 diabetes mellitus and the relevant factors. Chin. J. Gen. Surg..

[22] Enhörning S., Brunkwall L., Tasevska I., Ericson U., Tholin J.P., Persson M., Lemetais G., Vanhaecke T., Dolci A., Perrier E.T. (2019). Water supplementation reduces copeptin and plasma glucose in adults with high copeptin: The H_2_O metabolism pilot study. J. Clin. Endocrinol. Metab..

[23] Muscogiuri G., Barrea L., Annunziata G., Vecchiarini M., Orio F., Somma C.D., Colao A., Savastano S. (2018). Water intake keeps type 2 diabetes away? Focus on copeptin. Endocrine.

[24] Sedaghat G., Montazerifar F., Keykhaie M.A., Karajibani M., Shourestani S., Dashipour A. (2021). Effect of pre-meal water intake on the serum levels of copeptin, glycemic control, lipid profile and anthropometric indices in patients with type 2 diabetes mellitus: A randomized, controlled trial. J. Diabetes Metab. Disord..

[25] Korovljev D., Trivic T., Drid P., Ostojic S.M. (2018). Molecular hydrogen affects body composition, metabolic profiles, and mitochondrial function in middle-aged overweight women. Ir. J. Med. Sci..

[26] Wang N., Zhu F., Chen L., Chen K. (2018). Proteomics, metabolomics and metagenomics for type 2 diabetes and its complications. Life Sci..

[27] Miyamoto J., Igarashi M., Watanabe K., Karaki S.I., Mukouyama H., Kishino S., Li X., Ichimura A., Irie J., Sugimoto Y. (2019). Gut microbiota confers host resistance to obesity by metabolizing dietary polyunsaturated fatty acids. Nat. Commun..

[28] Huang F., Zheng X., Ma X., Jiang R., Zhou W., Zhou S., Zhang Y., Lei S., Wang S., Kuang J. (2019). Theabrownin from Pu-erh tea attenuates hypercholesterolemia via modulation of gut microbiota and bile acid metabolism. Nat. Commun..

[29] Pathak P., Xie C., Nichols R.G., Ferrell J.M., Boehme S., Krausz K.W., Patterson A.D., Gonzalez F.J., Chiang J.Y.L. (2018). Intestine farnesoid X receptor agonist and the gut microbiota activate G-protein bile acid receptor-1 signaling to improve metabolism. Hepatology.

[30] Dou Y., Ma C., Wang K., Liu S., Sun J., Tan W., Neckenig M., Wang Q., Dong Z., Gao W. (2022). Dysbiotic tumor microbiota associates with head and neck squamous cell carcinoma outcomes. Oral Oncol..

[31] Nistal E., Sáenz de Miera L.E., Ballesteros Pomar M., Sánchez-Campos S., García-Mediavilla M.V., Álvarez-Cuenllas B., Linares P., Olcoz J.L., Arias-Loste M.T., García-Lobo J.M. (2019). An altered fecal microbiota profile in patients with non-alcoholic fatty liver disease (NAFLD) associated with obesity. Rev. Esp. Enferm. Dig..

[32] Carbajo-Pescador S., Porras D., García-Mediavilla M.V., Martínez-Flórez S., Juarez-Fernández M., Cuevas M.J., Mauriz J.L., González-Gallego J., Nistal E., Sánchez-Campos S. (2019). Beneficial effects of exercise on gut microbiota functionality and barrier integrity, and gut-liver crosstalk in an in vivo model of early obesity and non-alcoholic fatty liver disease. Dis. Model. Mech..

[33] Oba P.M., Holscher H.D., Mathai R.A., Kim J., Swanson K.S. (2020). Diet Influences the oral microbiota of infants during the first six months of life. Nutrients.

[34] Inanova A.Y., Shirokov I.V., Toshchanko S.V., Kozlova A.D., Obolenskaya O.N., Mariasina S.S., Ivlev V.A., Gartseev I.B., Medvedev O.S. (2023). Effects of coenzyme Q10 on the biomarkers (hydrogen, methane, SCFA and TMA) and compositon of the gut microbiome in rats. Pharmaceuticals.

[35] Yang M., Dong Y., He Q., Zhu P., Zhuang Q., Shen J., Zhang X., Zhao M. (2020). Hydrogen: A novel option in human disease treatment. Oxid. Med. Cell. Longev..

