# Supplementary material for: Hydrogen-Rich Water Ameliorates Metabolic Disorder via Modifying Gut Microbiota in Impaired Fasting Glucose Patients: A Randomized Controlled Study
Source: Antioxidants (Basel). 2023 Jun 9;12(6):1245. doi: 10.3390/antiox12061245 (PMC10295603; doi:10.3390/antiox12061245)
Supplement: Supplementary file 1 [file antioxidants-12-01245-s001.zip › antioxidants-2374749-supplementary.pdf]

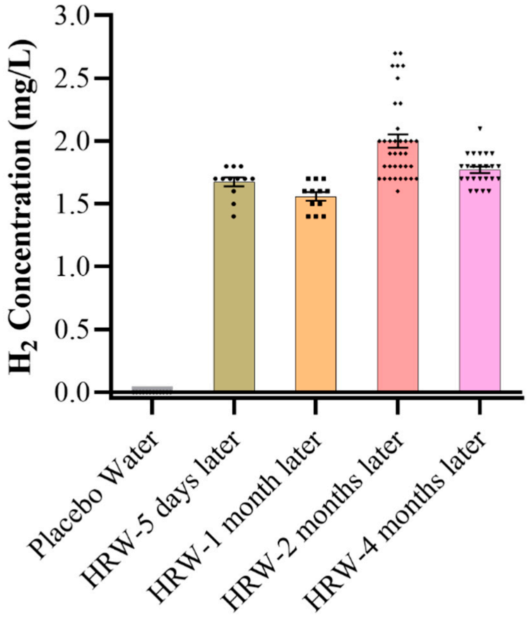

Figure S1. The hydrogen concentration (mg/L) of the experimental packaged Placebo water, and packaged HRW after production (5 days, 1 month, 2 months, 4 months).

Table S1. The baseline characteristics of the studied patients with IGF.

	Hydrogen-Rich Water Group (n=32)	Placebo Water Group (n=41)	*p*-Value	Test Method
**Male(%)**	46.30	50.00	0.756	(Pearson Chi-Square)
**Age (years)**	46.16±6.02	47.95±5.44	0.192	(Unpaired t test)
**Height (cm)**	168.95±8.02	168.06±7.79	0.638	(Unpaired t test)
**Weight (kg)**	69.50±19.78	68.10±20.30	0.640	(Mann Whitney test)
**Body mass index (kg/m^2)**	25.26±4.29	24.66±3.41	0.513	(Unpaired t test)
**Abdominal girth (cm)**	85.37±12.59	83.90±10.94	0.600	(Unpaired t test)

Mean ± SEM for continuous variables with a normal distribution; Median ± IR for non-normal continuous variables.

Table S2. The differential parameters between HRW group and placebo group after 8 weeks of treatment.

Variables	After 8 Weeks Treatment		*t*-Value	c-Value	*p*-Value
	Placebo Group	HRW Group			
Ala	335.06±56.04	338.48±59.64	0.25	.	0.8034
Arg	18.80±5.62	19.36±8.30	0.32	.	0.7476
Asn	105.47±22.62	103.14±20.06	-0.46	.	0.6501
Asp	66.76±14.79	68.07±13.06	0.39	.	0.6959
C102	0.05±0.02	0.04±0.01	.	0.3211	0.3211
C102C10	0.24±0.12	0.22±0.10	.	0.2766	0.2766
C142	0.12±0.04	0.13±0.04	.	0.3526	0.3526
C16	0.73±0.26	0.66±0.20	.	0.327	0.327
C5	0.17±0.11	0.14±0.05	.	0.5109	0.5109
C5DCC16	0.18±0.06	0.19±0.07	.	0.8428	0.8428
C5OHC8	1.62±0.74	1.55±0.73	-0.39	.	0.6965
Cit	21.94±3.62	20.41±4.78	-1.54	.	0.127
CitArg	1.26±0.41	1.26±0.67	.	0.4443	0.4443
Glu	170.20±332.29	121.33±19.79	.	0.5331	0.5331
GlyAla	0.66±0.20	0.62±0.15	.	0.8295	0.8295
MetLeu	0.13±0.02	0.14±0.02	0.77	.	0.4455
MetPhe	0.36±0.05	0.37±0.04	0.23	.	0.8154
Orn	32.34±5.09	30.99±6.59	.	0.1775	0.1775
OrnCit	1.49±0.21	1.55±0.26	1.05	.	0.2983
PheTyr	0.98±0.13	0.99±0.12	0.44	.	0.6614
Pip	941.73±630.14	839.89±429.77	.	0.8918	0.8918
Thr	40.88±9.15	43.64±10.54	1.19	.	0.2388
TyrCit	2.27±0.53	2.48±0.61	.	0.2043	0.2043
ValPhe	3.01±0.49	3.02±0.47	0.12	.	0.9026

Data with t-Value follows normal distribution, compared by *t* test; data with c-Value were non-normal variables, were compared by Mann-Whitney test. * *P* was significant at 0.002 (0.05/24) after Bonferroni correction.

Table S3. The differential parameters between pre- and post-treatment in placebo group.

Variables	Placebo Water Group (n=41)		*t*-Value	c-Value	*p*-Value
	0 week	8 weeks			
Ala	335.06±56.04	319.08±68.17	1.6	.	0.1183
Arg	18.80±5.62	15.59±5.00**	3.11	.	0.0035
Asn	105.47±22.62	96.46±22.51*	2.52	.	0.0161
Asp	66.76±14.79	69.74±22.81	-1.08	.	0.2878
C102	0.05±0.02	0.05±0.02	.	0.4076	0.4076
C102C10	0.24±0.12	0.24±0.14	.	0.7254	0.7254
C14：2	0.12±0.04	0.13±0.04	.	0.2161	0.2161
C16	0.73±0.26	0.76±0.27	.	0.5067	0.5067
C5	0.17±0.11	0.17±0.09	.	0.4442	0.4442
C5DCC16	0.18±0.06	0.17±0.05	.	0.5899	0.5899
C5OHC8	1.62±0.74	1.59±0.92	0.22	.	0.824
Cit	21.94±3.62	21.02±4.61	1.41	.	0.1663
CitArg	1.26±0.41	1.44±0.42*	.	0.0232	0.0232
Glu	170.20±332.29	112.77±15.83	.	0.2006	0.2006
GlyAla	0.66±0.20	0.60±0.16	.	0.1658	0.1658
MetLeu	0.13±0.02	0.12±0.03*	2.06	.	0.0461
MetPhe	0.36±0.05	0.34±0.05***	3.89	.	0.0004
Orn	32.34±5.09	30.76±12.82*	.	0.0117	0.0117
OrnCit	1.49±0.21	1.45±0.37	0.65	.	0.5209
PheTyr	0.98±0.13	0.99±0.13	-0.39	.	0.6977
Pip	941.73±630.14	825.54±491.72	.	0.4705	0.4705
Thr	40.88±9.15	36.72±8.18*	2.61	.	0.0127
TyrCit	2.27±0.53	2.27±0.61	.	0.8136	0.8136
ValPhe	3.01±0.49	2.98±0.43	0.51	.	0.6162

Data with t-Value follows normal distribution, compared by t test; data with c-Value were non-normal variables, were compared by Mann-Whitney test (*: p≤0.05; **: p≤0.01; ***: P≤0.001). * *P* was significant at 0.002 (=0.05/24) after Bonferroni correction.

Table S4. The differential parameters between pre- and post-treatment in HRW group.

Variables	Hydrogen-Rich Water Group (n=32)		*t*-Value	c-Value	*p*-Value
	0 week	8 weeks			
Ala	338.48±59.64	318.61±65.22	1.63	.	0.1139
Arg	19.36±8.30	16.06±7.65	.	0.0583	0.0583
Asn	103.14±20.06	94.54±19.01	.	0.0548	0.0548
Asp	68.07±13.06	66.71±14.95	0.59	.	0.558
C102	0.04±0.01	0.04±0.01	1.99	.	0.056
C102C10	0.22±0.10	0.23±0.13	.	0.727	0.727
C14：2	0.13±0.04	0.11±0.03*	.	0.0333	0.0333
C16	0.66±0.20	0.68±0.18	.	0.6052	0.6052
C5	0.14±0.05	0.14±0.04	0.44	.	0.6626
C5DCC16	0.19±0.07	0.16±0.05	.	0.11	0.11
C5OHC8	1.55±0.73	1.62±1.11	.	0.6674	0.6674
Cit	20.41±4.78	18.19±3.99***	3.81	.	0.0006
CitArg	1.26±0.67	1.31±0.46	.	0.2295	0.2295
Glu	121.33±19.79	108.21±14.59**	.	0.0041	0.0041
GlyAla	0.62±0.15	0.59±0.17	.	0.2294	0.2294
MetLeu	0.14±0.02	0.13±0.02	1.66	.	0.1079
MetPhe	0.37±0.04	0.36±0.06	0.96	.	0.3431
Orn	30.99±6.59	26.95±5.73*	.	0.0195	0.0195
OrnCit	1.55±0.26	1.52±0.39	.	0.3473	0.3473
PheTyr	0.99±0.12	0.98±0.14	0.64	.	0.5257
Pip	839.89±429.77	780.22±401.00	.	0.3473	0.3473
Thr	43.64±10.54	37.19±7.44**	3.62	.	0.001
TyrCit	2.48±0.61	2.61±0.75	.	0.5866	0.5866
ValPhe	3.02±0.47	3.06±0.49	-0.36	.	0.7186

Data with t-Value follows normal distribution, compared by *t* test; data with c-Value were non-normal variables, were compared by Mann-Whitney test (*: p≤0.05; **: p≤0.01; ***: p≤0.001). * *P* was significant at 0.002 (=0.05/24) after Bonferroni correction.

Table S5. The Pearson correlation analysis of gut flora and metabolites.

	C5	Asn	Trp	Ser	C16	Lys	C8.C10	C18.2	C5DC	C18	C5.C0	C5.1	C5.OH	C4	C18.1	C5.C2	Val.Phe	C6	C10.2	C5.C3	C4.OH	C8	Val	C0	C10.1	Gln	C22	C2	Pro	Pip	C18.OH	C18.1.OH	Gly	C26	C16.1.OH	His	Eubacterium	Acetobacter	Acidovorax	Cetobacteriu	Dechloromo	Dechlorosor	Dorea	Dubosiella	Flavobacteri	Hydrogenop	Hyphomicro	Lactobacillu	Lactococcus	Megasphaer	Methylovers	MND1	Negativibac	Persicirhabd	Prevotella_9	Reyranella	Ruminiclost	Ruminococ	Simplicispira	Terrimonas	Thauera	Tyzzerella_4	uncultured_	uncultured_	uncultured_	uncultured_
C5	0	0.00086796	0.00431005	0.01629208	0.01341793	0.01398091	0.02981987	0.05280778	0.00025063	0.03742251	0.00097621	0.11609372	0.00509033	0.00055709	0.08771468	0.00216697	0.00631712	0.00172461	0.02235753	0.00513423	0.18005609	0.00506113	0.0308557	0.03826738	0.03278408	0.09772612	0.12080671	0.10526235	0.03458859	0.18513893	0.0477397	0.10936346	0.15749327	0.07694858	0.14813583	0.3044568	0.18228263	0.00603008	0.07236056	0.2145281	0.00604858	0.01135974	0.03218849	0.21106658	0.03336981	0.01847368	0.01917234	0.20138784	0.33088173	0.07984431	0.00892593	0.27566066	0.04081232	1.24E-05	0.23831346	0.20603347	0.29003828	0.12344562	0.01421485	0.00564642	0.01175824	0.09464285	0.15595886	0.09342012	0.0344777	0.01529019
Asn	0.00086796	0	0.00871311	0.02305713	0.04468894	0.01912198	0.01406313	0.05248947	0.01066897	0.07710004	0.00067983	0.03446957	0.00436168	0.00649806	0.16888646	0.00260035	0.03327151	0.00594657	0.00632578	0.00909983	0.14106873	0.04168077	0.00833016	0.17182834	0.14088335	0.13806993	0.09894297	0.27612573	0.18533969	0.0614427	0.02348584	0.12900163	0.0955	0.1601194	0.49491245	0.22446991	0.19467901	0.01601961	0.05136425	0.1944627	0.00522169	0.01296786	0.10813058	0.09985848	0.01118096	0.0263265	0.01212211	0.09957668	0.22521649	0.17306889	0.00479996	0.17756227	0.052401	0.00047177	0.12785056	0.10407837	0.43144507	0.05532115	0.00335623	0.02408053	0.02378284	0.12020964	0.07296765	0.19871617	0.10395887	0.01103504
Trp	0.00431005	0.00871311	0	0.00627952	0.00630516	0.04313733	0.00084565	0.00412721	0.00927991	0.01599402	0.05837665	0.02355139	0.1160248	0.0528946	0.02253207	0.11697669	0.13960325	0.08021678	0.21010884	0.12772478	0.0738095	0.09149094	0.05453026	0.01026988	0.05339357	0.24066212	0.02624045	0.05626115	0.01045825	0.05739583	0.34578373	0.02634742	0.08445977	0.22054913	0.0217101	0.26777797	0.09294894	0.00169369	0.01712172	0.11449922	0.00201351	0.00491966	0.02076415	0.05534123	0.00922411	0.01327023	0.00245122	0.18630113	0.15723382	0.09902155	0.00269123	0.27024557	0.31035211	0.00061906	0.05713795	0.07997896	0.34688334	0.11131207	0.00193833	0.00989269	0.01449566	0.29110698	0.00546505	0.16331222	0.03015887	0.00328617
Ser	0.01629208	0.02305713	0.00627952	0	0.00891591	0.00017332	0.00693624	0.00204505	0.04851759	0.0101779	0.06445667	0.00931747	0.06235376	0.11138463	0.02899097	0.09147099	0.12259504	0.13053448	0.06273893	0.1202174	0.0020972	0.16202649	0.25741866	0.12859719	0.06780224	0.01206928	0.07934021	0.11577555	0.20910392	0.09098316	0.28403416	0.36714315	0.2574051	0.29397835	0.08295975	0.16673858	0.11083471	0.00013693	0.00261836	0.1082537	5.49E-05	0.00049503	0.01144817	0.12249884	0.01585619	0.00699362	0.00095892	0.34782347	0.10141954	0.05148182	0.00159515	0.04415948	0.02349693	0.00226094	0.09527641	0.08582551	0.1231689	0.16310886	0.00271627	0.00260321	0.00397823	0.06248793	0.01651747	0.13181208	0.01215321	4.30E-05
C16	0.01341793	0.04468894	0.00630516	0.00891591	0	0.01750641	0.00491455	0.00104204	0.06621972	0.0004437	0.07396645	0.01010967	0.10710837	0.16699375	0.00031028	0.23841336	0.05042068	0.25654394	0.25735185	0.32358839	0.02650431	0.1046992	0.08688127	0.04110995	0.03638674	0.09497499	0.1346125	0.00217685	0.04109882	0.16514723	0.3639245	0.07606385	0.11355136	0.07398508	0.03003122	0.10128887	0.20193477	0.0011794	0.17153394	0.23522089	0.015375	0.02253967	0.00126235	0.05378529	0.01667149	0.02224657	0.0248529	0.28564497	0.36846491	0.10417424	0.02024792	0.44915056	0.19605536	0.01190541	0.43957463	0.33492921	0.43800089	0.03950653	0.03312172	0.09539418	0.05923211	0.07629945	0.12415343	0.11553947	0.01971714	0.02435948
Lys	0.01398091	0.01912198	0.04313733	0.00017332	0.01750641	0	0.03229189	0.01171769	0.04189651	0.03436591	0.03262799	0.01643657	0.02140839	0.08813332	0.04332502	0.04418768	0.05843437	0.07031131	0.01145178	0.04636545	0.00364222	0.0722378	0.18986124	0.22625853	0.04642075	0.0002663	0.13679155	0.11195482	0.33295323	0.23798	0.13374081	0.56042184	0.44536431	0.29648785	0.25792308	0.10495215	0.3023552	0.00426233	0.03130381	0.18241785	0.00205627	0.00579723	0.02238056	0.16490401	0.04428537	0.02959399	0.01520635	0.52422707	0.11722372	0.14891761	0.01876835	0.03781262	0.00194759	0.00547083	0.35114747	0.16063881	0.19544249	0.09675477	0.02241629	0.01381969	0.02198806	0.00953066	0.09362744	0.26102194	0.05211235	0.00178609
C8.C10	0.02981987	0.01406313	0.00084565	0.00693624	0.00491455	0.03229189	0	0.00020276	0.05576353	0.03106883	0.11820574	0.00298906	0.25865029	0.13867832	0.00608305	0.22922992	0.32320489	0.26213174	0.1787013	0.32037176	0.01337048	0.23990906	0.09708785	0.04315229	0.11839788	0.18962373	0.02838657	0.05805116	0.05814131	0.04905722	0.26397521	0.0151889	0.01706027	0.49599191	0.11616502	0.05649937	0.26012715	0.00486661	0.03704658	0.02922819	0.00303751	0.00681335	0.00652154	0.00206938	0.02961019	0.01412032	0.01038379	0.13586852	0.03906308	0.17130117	0.00712215	0.09682312	0.32282409	0.00930765	0.1634011	0.01467238	0.30882065	0.10257662	0.00081648	0.05139947	0.01661828	0.2568129	0.00378645	0.21404066	0.04179936	0.0090642
C18.2	0.05280778	0.05248947	0.00412721	0.00204505	0.00104204	0.01171769	0.00020276	0	0.09723325	0.0061685	0.22942099	0.00052022	0.2490073	0.27384606	0.00040713	0.3605785	0.33545045	0.4251397	0.29212411	0.40310824	0.00508073	0.32706139	0.19868489	0.03450152	0.12340433	0.07093861	0.01610093	0.01834483	0.08809671	0.05352437	0.34895981	0.06796202	0.10407279	0.35653497	0.049363	0.01968158	0.36972087	0.00154335	0.06571969	0.13025619	0.00797744	0.02041636	0.00083548	0.01964693	0.0345364	0.04779683	0.0221554	0.20964874	0.09469283	0.23775631	0.01848527	0.14588176	0.23348928	0.0204967	0.27134454	0.07590351	0.46821463	0.08738353	0.01157363	0.06434443	0.03875585	0.07534258	0.01824479	0.30781295	0.0631989	0.0112197
C5DC	0.00025063	0.01066897	0.00927991	0.04851759	0.06621972	0.04189651	0.05576353	0.09723325	0	0.18659311	0.02110842	0.25304516	0.04865059	0.00072466	0.12486452	0.01027134	0.06148627	0.00185675	0.04966447	0.00369446	0.28390412	0.00363514	0.07287686	0.00971243	0.02572019	0.12102455	0.06244351	0.12782797	0.00982191	0.37710242	0.07135607	0.0800163	0.27863308	0.32362449	0.13641664	0.29728003	0.46421259	0.04414031	0.11050486	0.17391512	0.02666113	0.04172536	0.05298334	0.23947746	0.18118336	0.08280452	0.08289384	0.38836485	0.16848325	0.25493982	0.06532091	0.20858715	0.10796966	0.00087974	0.38929171	0.12807609	0.34529006	0.26848913	0.04768495	0.00755209	0.0296392	0.16308885	0.14450415	0.30643112	0.12531159	0.04692759
C18	0.03742251	0.07710004	0.01599402	0.0101779	0.0004437	0.03436591	0.03106883	0.0061685	0.18659311	0	0.11427231	0.01507325	0.07098392	0.27199526	0.01340578	0.2698181	0.06325263	0.37176582	0.42082478	0.4014804	0.0723919	0.31246436	0.19040782	0.11214468	0.18490755	0.16613905	0.16719398	0.03446792	0.16796543	0.05013528	0.46407423	0.25733927	0.22551929	0.01297044	0.02927211	0.23052477	0.0861879	0.00036729	0.12902557	0.59574501	0.02582235	0.04331248	0.01234771	0.27518124	0.00449058	0.04548089	0.01734561	0.16909124	0.81309858	0.06428326	0.01010865	0.70723332	0.18063284	0.02452589	0.18442973	0.61575069	0.58161184	0.08373674	0.05080176	0.08622859	0.0751697	0.0752232	0.18945248	0.07938893	0.02760818	0.02656484
C5.C0	0.00097621	0.00067983	0.05837665	0.06445667	0.07396645	0.03262799	0.11820574	0.22942099	0.02110842	0.11427231	0	0.19150198	0.00076076	0.00470699	0.33810052	0.00054925	0.00080979	0.00120985	0.00237925	0.00658376	0.24764103	0.00844018	0.01509429	0.39774147	0.07904348	0.14617699	0.52016298	0.3453992	0.27941377	0.30950791	0.05186825	0.37277996	0.21801316	0.07923281	0.62039553	0.62999884	0.1064338	0.04562136	0.11881721	0.29199573	0.01913258	0.02036468	0.23384906	0.28917492	0.02515006	0.01883297	0.02669642	0.29830581	0.51717495	0.07690445	0.01781087	0.3358482	0.02447718	0.00291404	0.26302911	0.37538516	0.26147293	0.08749186	0.03080007	0.04696003	0.04892686	0.1818963	0.29949797	0.08767015	0.06140057	0.03259028
C5.1	0.11609372	0.03446957	0.02355139	0.00931747	0.01010967	0.01643657	0.00298906	0.00052022	0.25304516	0.01507325	0.19150198	0	0.15350929	0.47364554	0.0097118	0.38059804	0.32114146	0.45821991	0.21145762	0.39531181	0.00750761	0.42498672	0.08361937	0.21466629	0.22843837	0.07060081	0.04066348	0.06002131	0.34323541	0.04020742	0.35253328	0.20423997	0.21558635	0.32403612	0.25280418	0.02972909	0.37115412	0.01222061	0.10848521	0.31235494	0.02222954	0.04978608	0.03782222	0.02622528	0.00942208	0.10796232	0.02739579	0.25095979	0.16969872	0.45555311	0.03280241	0.21986031	0.22284613	0.05305911	0.26159238	0.172094	0.81245153	0.01307817	0.02539547	0.19266319	0.15451831	0.05441796	0.02719607	0.57691048	0.20956728	0.01525762
C5.OH	0.00509033	0.00436168	0.1160248	0.06235376	0.10710837	0.02140839	0.25865029	0.2490073	0.04865059	0.07098392	0.00076076	0.15350929	0	0.02255672	0.40078001	0.00141874	0.00177297	0.00464927	0.01101063	0.00284639	0.33983411	0.03600094	0.03728968	0.43806276	0.19063986	0.07221676	0.36056622	0.39034906	0.49231929	0.2041128	0.04241998	0.73036994	0.62474766	0.0204495	0.60938349	0.51566371	0.20313647	0.04447918	0.18048882	0.85662187	0.05419754	0.08692968	0.28056681	0.69994032	0.02319303	0.12890559	0.06097111	0.29246856	0.88944534	0.20706139	0.04143112	0.5282537	0.00684523	0.00950351	0.28199104	0.73516397	0.65823757	0.07352393	0.11036291	0.05920733	0.12907425	0.03037299	0.49495006	0.24346184	0.20660254	0.04963421
C4	0.00055709	0.00649806	0.0528946	0.11138463	0.16699375	0.08813332	0.13867832	0.27384606	0.00072466	0.27199526	0.00470699	0.47364554	0.02255672	0	0.41533403	0.00020601	0.0418026	0.00081245	0.02050311	0.00247697	0.5249392	0.01976351	0.13620178	0.09453875	0.16982734	0.26652591	0.21638529	0.48849725	0.0999388	0.2860139	0.01059825	0.1608231	0.15360729	0.23186683	0.45106903	0.4745207	0.38521924	0.07304068	0.13267977	0.20979882	0.04401216	0.05918912	0.12745162	0.44040207	0.23868346	0.06986394	0.11667127	0.1511684	0.36521669	0.12594581	0.04752283	0.23037351	0.05590299	0.00289642	0.30715136	0.15514932	0.23696017	0.5275436	0.04554608	0.00935242	0.01412051	0.25534965	0.35583414	0.10654879	0.10220982	0.10581306
C18.1	0.08771468	0.16888646	0.02253207	0.02899097	0.00031028	0.04332502	0.00608305	0.00040713	0.12486452	0.01340578	0.33810052	0.0097118	0.40078001	0.41533403	0	0.61437777	0.2800938	0.56157978	0.49528623	0.59044088	0.01932142	0.21272342	0.18523546	0.02383226	0.04629056	0.09403461	0.07029972	0.00019267	0.03426355	0.30521866	0.57019354	0.05520043	0.19496112	0.34274571	0.02903761	0.02751126	0.60017054	0.01908512	0.3262292	0.21458749	0.06591248	0.09044683	0.00101388	0.02349776	0.10910678	0.11825724	0.11840452	0.51562701	0.18662914	0.40193131	0.12965087	0.38723205	0.42637837	0.07333708	0.79613885	0.26392551	0.65263362	0.06012382	0.10627107	0.23664508	0.17418498	0.09247892	0.11081628	0.44276412	0.11220626	0.07882998
C5.C2	0.00216697	0.00260035	0.11697669	0.09147099	0.23841336	0.04418768	0.22922992	0.3605785	0.01027134	0.2698181	0.00054925	0.38059804	0.00141874	0.00020601	0.61437777	0	0.0173313	0.00025582	0.00190865	0.0002974	0.45202743	0.02897175	0.10646424	0.34959483	0.24022377	0.1475598	0.3452648	0.70777868	0.38528768	0.2563123	0.00610862	0.49296124	0.31916785	0.1658189	0.78497208	0.55940719	0.29980365	0.08212906	0.11023391	0.37313932	0.04027703	0.05801571	0.27050537	0.60082844	0.1396243	0.0819498	0.0875481	0.19348875	0.49249383	0.14095854	0.03940905	0.21029844	0.00808415	0.00513679	0.25173121	0.27167781	0.28232761	0.36721304	0.05456086	0.01562349	0.02986214	0.14300921	0.42476786	0.14440919	0.14996056	0.07222923
Val.Phe	0.00631712	0.03327151	0.13960325	0.12259504	0.05042068	0.05843437	0.32320489	0.33545045	0.06148627	0.06325263	0.00080979	0.32114146	0.00177297	0.0418026	0.2800938	0.0173313	0	0.01267111	0.0416489	0.03368446	0.35154577	0.00567109	0.03596297	0.43122089	0.04064911	0.13606863	0.83538288	0.16633208	0.26466244	0.63984192	0.21790612	0.63242446	0.5683815	0.01367953	0.38996752	0.8547169	0.11114632	0.07057845	0.34128039	0.6957424	0.08801561	0.081764	0.2603783	0.6011833	0.04065935	0.06241395	0.08192978	0.64604665	0.96056011	0.09374815	0.08328045	0.81725504	0.04365069	0.02228206	0.56278133	0.97672964	0.41857735	0.07117802	0.19481808	0.13896282	0.18879323	0.13547789	0.67502385	0.10544732	0.07859457	0.09499292
C6	0.00172461	0.00594657	0.08021678	0.13053448	0.25654394	0.07031131	0.26213174	0.4251397	0.00185675	0.37176582	0.00120985	0.45821991	0.00464927	0.00081245	0.56157978	0.00025582	0.01267111	0	0.00582431	2.43E-05	0.50537475	0.001789	0.03843582	0.23265868	0.06725803	0.15583047	0.34616885	0.4841489	0.17255716	0.53437957	0.05461699	0.42824864	0.54751587	0.23781828	0.57666643	0.77254648	0.29924336	0.14302301	0.14253958	0.38899619	0.05875161	0.07072135	0.38302549	0.55394651	0.1451552	0.11353361	0.09703606	0.5361876	0.42240792	0.25606578	0.0910394	0.31462597	0.04277392	0.00525387	0.334337	0.35642409	0.36785099	0.23700682	0.09996114	0.02629612	0.08936224	0.22600296	0.33231022	0.32576585	0.22315701	0.07087578
C10.2	0.02235753	0.00632578	0.21010884	0.06273893	0.25735185	0.01145178	0.1787013	0.29212411	0.04966447	0.42082478	0.00237925	0.21145762	0.01101063	0.02050311	0.49528623	0.00190865	0.0416489	0.00582431	0	0.00544598	0.12340066	0.03071578	0.09735099	0.69513727	0.13037353	0.03031367	0.50934259	0.62654557	0.65988575	0.51766589	0.02820359	0.71533512	0.40831448	0.53215852	0.8753286	0.40312225	0.41160174	0.14347243	0.10518205	0.16165056	0.02907126	0.03346426	0.32825042	0.25800232	0.16116106	0.06304701	0.08327805	0.56182856	0.15240635	0.25297765	0.07741988	0.04142963	0.00321836	0.02281396	0.47344372	0.15941825	0.17364625	0.19200691	0.05422765	0.06209142	0.06464991	0.12446896	0.27629263	0.32653101	0.18460513	0.04218239
C5.C3	0.00513423	0.00909983	0.12772478	0.1202174	0.32358839	0.04636545	0.32037176	0.40310824	0.00369446	0.4014804	0.00658376	0.39531181	0.00284639	0.00247697	0.59044088	0.0002974	0.03368446	2.43E-05	0.00544598	0	0.50834928	0.00946786	0.07269496	0.24469728	0.1359602	0.08183569	0.21315385	0.55668599	0.28694092	0.44559049	0.02341451	0.58061072	0.75558775	0.26627429	0.68217776	0.53707149	0.52982563	0.15741592	0.17057891	0.57207959	0.0843253	0.12688174	0.37167444	0.70492972	0.19907132	0.24038673	0.15719011	0.49691983	0.42870352	0.44246604	0.13386026	0.26170612	0.01809555	0.01032674	0.4000652	0.37561147	0.5573911	0.25867076	0.14845663	0.02800128	0.11995742	0.08929815	0.40194935	0.53595622	0.39238664	0.08820506
C4.OH	0.18005609	0.14106873	0.0738095	0.0020972	0.02650431	0.00364222	0.01337048	0.00508073	0.28390412	0.0723919	0.24764103	0.00750761	0.33983411	0.5249392	0.01932142	0.45202743	0.35154577	0.50537475	0.12340066	0.50834928	0	0.33002642	0.38044721	0.40194873	0.07127676	0.01325094	0.26100549	0.09536149	0.43641672	0.3556698	0.65009923	0.49773005	0.3102529	0.81531983	0.24841533	0.12616793	0.27800801	0.02304042	0.03765915	0.04736366	0.00723207	0.00755233	0.03783264	0.02481201	0.07820911	0.02400196	0.02006229	0.81272917	0.0295174	0.18640618	0.04865897	0.02414212	0.10812021	0.08001675	0.39503437	0.08550422	0.12324603	0.12985744	0.02677426	0.11291931	0.06553246	0.15439839	0.02208792	0.35013201	0.04801404	0.0055457
C8	0.00506113	0.04168077	0.09149094	0.16202649	0.1046992	0.0722378	0.23990906	0.32706139	0.00363514	0.31246436	0.00844018	0.42498672	0.03600094	0.01976351	0.21272342	0.02897175	0.00567109	0.001789	0.03071578	0.00946786	0.33002642	0	0.02262939	0.15639449	0.00146655	0.09848614	0.51120695	0.09388057	0.05160633	0.92407113	0.24275264	0.32465736	0.65344187	0.28097685	0.32786412	0.68668144	0.40633116	0.18464562	0.35487346	0.32452028	0.10290202	0.09348547	0.25185018	0.31788949	0.19469985	0.11658387	0.16159423	0.91197995	0.37020058	0.34695357	0.22578708	0.46469351	0.12066827	0.01936143	0.86050424	0.55023786	0.3919767	0.09668961	0.22043796	0.11847925	0.22291137	0.22448596	0.39968623	0.43473932	0.19396492	0.11056109
Val	0.0308557	0.00833016	0.05453026	0.25741866	0.08688127	0.18986124	0.09708785	0.19868489	0.07287686	0.19040782	0.01509429	0.08361937	0.03728968	0.13620178	0.18523546	0.10646424	0.03596297	0.03843582	0.09735099	0.07269496	0.38044721	0.02262939	0	0.27383747	0.07528157	0.35914881	0.30005714	0.1125539	0.14310655	0.39247429	0.25805582	0.14209788	0.36016688	0.1627239	0.57059739	0.45745241	0.3484594	0.19602783	0.4660525	0.58285689	0.15083375	0.17886517	0.41065376	0.11311806	0.02417937	0.20452518	0.12276162	0.52418436	0.54550572	0.67177778	0.16128915	0.84542445	0.42433484	0.04738771	0.53107959	0.58593429	0.86379234	0.00148168	0.14674174	0.40200747	0.47574467	0.27518742	0.22416652	0.71731462	0.46280477	0.13262589
C0	0.03826738	0.17182834	0.01026988	0.12859719	0.04110995	0.22625853	0.04315229	0.03450152	0.00971243	0.11214468	0.39774147	0.21466629	0.43806276	0.09453875	0.02383226	0.34959483	0.43122089	0.23265868	0.69513727	0.24469728	0.40194873	0.15639449	0.27383747	0	0.12474402	0.40605149	0.00877381	0.03067077	0.0003342	0.25790944	0.35743379	0.00998823	0.24573215	0.38734514	0.0093304	0.13627402	0.7873926	0.06335101	0.32403977	0.34999681	0.15005026	0.22027331	0.01247808	0.22789503	0.3367245	0.30384628	0.25466864	0.29809253	0.29889565	0.52063419	0.19633942	0.58629415	0.66642353	0.04262579	0.53971353	0.21497563	0.8301106	0.41913113	0.16241104	0.08133661	0.13100985	0.27043913	0.16717551	0.52333109	0.25894354	0.20214574
C10.1	0.03278408	0.14088335	0.05339357	0.06780224	0.03638674	0.04642075	0.11839788	0.12340433	0.02572019	0.18490755	0.07904348	0.22843837	0.19063986	0.16982734	0.04629056	0.24022377	0.04064911	0.06725803	0.13037353	0.1359602	0.07127676	0.00146655	0.07528157	0.12474402	0	0.05643901	0.48658477	0.01393713	0.02990852	0.83551924	0.76655388	0.31593661	0.67354333	0.48870156	0.09248546	0.60406634	0.28904888	0.11467359	0.232355	0.16103694	0.05919675	0.04197827	0.1309382	0.13093734	0.15306916	0.06117699	0.0860173	0.57219942	0.17663948	0.27863226	0.20729746	0.34573394	0.24015424	0.03940483	0.83140357	0.45062338	0.24168542	0.07522445	0.1818868	0.14161418	0.22250352	0.29699766	0.15810288	0.45626287	0.09309736	0.04765019
Gln	0.09772612	0.13806993	0.24066212	0.01206928	0.09497499	0.0002663	0.18962373	0.07093861	0.12102455	0.16613905	0.14617699	0.07060081	0.07221676	0.26652591	0.09403461	0.1475598	0.13606863	0.15583047	0.03031367	0.08183569	0.01325094	0.09848614	0.35914881	0.40605149	0.05643901	0	0.25570843	0.13971032	0.56192458	0.6736033	0.24580856	0.99534266	0.97627069	0.5522916	0.44272309	0.11719226	0.73332684	0.07494645	0.17136869	0.38675242	0.05377185	0.07937913	0.08857959	0.34420781	0.22404189	0.20375811	0.1451031	0.91252397	0.15379524	0.49195152	0.20365195	0.06045455	0.00417426	0.07559445	0.86530241	0.3714714	0.36583748	0.13889007	0.2074632	0.09808053	0.17337424	0.00461453	0.2878062	0.71104653	0.25638507	0.03574954
C22	0.12080671	0.09894297	0.02624045	0.07934021	0.1346125	0.13679155	0.02838657	0.01610093	0.06244351	0.16719398	0.52016298	0.04066348	0.36056622	0.21638529	0.07029972	0.3452648	0.83538288	0.34616885	0.50934259	0.21315385	0.26100549	0.51120695	0.30005714	0.00877381	0.48658477	0.25570843	0	0.18767606	0.09877128	0.03681457	0.17994912	0.06546723	0.33825406	0.62462252	0.14613087	0.02169929	0.96379804	0.07335639	0.19177865	0.48266335	0.12919124	0.26640148	0.04521053	0.20777245	0.24986716	0.51347671	0.22974406	0.13546905	0.16256274	0.93137644	0.16360775	0.27709706	0.47278225	0.07140571	0.30143773	0.08891883	0.81299777	0.32024725	0.10542295	0.09560293	0.16543404	0.10734508	0.06732214	0.99551926	0.61655924	0.13450071
C2	0.10526235	0.27612573	0.05626115	0.11577555	0.00217685	0.11195482	0.05805116	0.01834483	0.12782797	0.03446792	0.3453992	0.06002131	0.39034906	0.48849725	0.00019267	0.70777868	0.16633208	0.4841489	0.62654557	0.55668599	0.09536149	0.09388057	0.1125539	0.03067077	0.01393713	0.13971032	0.18767606	0	0.01755513	0.69207591	0.80622022	0.09828045	0.47272192	0.25116042	0.02548493	0.12369462	0.66213136	0.07956618	0.63944071	0.46491182	0.19226392	0.21663483	0.01853874	0.08535566	0.15637698	0.24231781	0.24230923	0.92918475	0.42125578	0.58842807	0.31925143	0.81182189	0.59046012	0.13000776	0.86813922	0.67391114	0.91902442	0.03279713	0.32322573	0.42875928	0.44460177	0.12822579	0.28341557	0.64155502	0.22367507	0.1831885
Pro	0.03458859	0.18533969	0.01045825	0.20910392	0.04109882	0.33295323	0.05814131	0.08809671	0.00982191	0.16796543	0.27941377	0.34323541	0.49231929	0.0999388	0.03426355	0.38528768	0.26466244	0.17255716	0.65988575	0.28694092	0.43641672	0.05160633	0.14310655	0.0003342	0.02990852	0.56192458	0.09877128	0.01755513	0	0.57816163	0.61491677	0.00684023	0.22258538	0.41707196	0.01064542	0.40365547	0.52536115	0.11611468	0.38156598	0.23129672	0.16184742	0.17384437	0.04729999	0.15119912	0.30033605	0.18517844	0.21956313	0.58577521	0.31280932	0.39823778	0.22835003	0.74314619	0.8695373	0.0490302	0.62484397	0.29624985	0.62660821	0.31799577	0.18016808	0.14218244	0.1865869	0.5808708	0.1663266	0.41924006	0.17468272	0.21453251
Pip	0.18513893	0.0614427	0.05739583	0.09098316	0.16514723	0.23798	0.04905722	0.05352437	0.37710242	0.05013528	0.30950791	0.04020742	0.2041128	0.2860139	0.30521866	0.2563123	0.63984192	0.53437957	0.51766589	0.44559049	0.3556698	0.92407113	0.39247429	0.25790944	0.83551924	0.6736033	0.03681457	0.69207591	0.57816163	0	0.17524615	0.2119721	0.09470236	0.19411362	0.39381823	0.18690344	0.28760765	0.03024632	0.09282275	0.64040136	0.08860768	0.17625272	0.14681864	0.37991888	0.04923464	0.23510112	0.07630742	0.00168437	0.6645555	0.28519548	0.01851076	0.49184492	0.40258025	0.09556153	0.02202149	0.19932298	0.93108588	0.43474943	0.03133408	0.11523475	0.08163884	0.28959492	0.12425769	0.26565682	0.28769341	0.11372869
C18.OH	0.0477397	0.02348584	0.34578373	0.28403416	0.3639245	0.13374081	0.26397521	0.34895981	0.07135607	0.46407423	0.05186825	0.35253328	0.04241998	0.01059825	0.57019354	0.00610862	0.21790612	0.05461699	0.02820359	0.02341451	0.65009923	0.24275264	0.25805582	0.35743379	0.76655388	0.24580856	0.17994912	0.80622022	0.61491677	0.17524615	0	0.33072357	0.21035155	0.35307838	0.77214825	0.0981854	0.84307761	0.23460901	0.49264479	0.53556023	0.23199229	0.35187214	0.19963415	0.52689444	0.49350443	0.42099951	0.48676286	0.05021048	0.49178014	0.61427632	0.21404285	0.23420174	0.04484411	0.0836915	0.7254722	0.21790178	0.78076045	0.5285433	0.18143039	0.15633936	0.12188822	0.0733428	0.77655746	0.43766815	0.55123754	0.38931703
C18.1.OH	0.10936346	0.12900163	0.02634742	0.36714315	0.07606385	0.56042184	0.0151889	0.06796202	0.0800163	0.25733927	0.37277996	0.20423997	0.73036994	0.1608231	0.05520043	0.49296124	0.63242446	0.42824864	0.71533512	0.58061072	0.49773005	0.32465736	0.14209788	0.00998823	0.31593661	0.99534266	0.06546723	0.09828045	0.00684023	0.2119721	0.33072357	0	0.01139348	0.62991016	0.17214077	0.14630216	0.78737902	0.18634562	0.51746194	0.13636896	0.23174529	0.2724473	0.05534216	0.02649046	0.35213133	0.23572728	0.32692938	0.12035938	0.22837525	0.57016305	0.2164311	0.63737251	0.83604311	0.12321916	0.58374485	0.09368735	0.84107377	0.35827137	0.10389328	0.34500186	0.19651774	0.77951523	0.14826991	0.43633977	0.29908426	0.38780541
Gly	0.15749327	0.0955	0.08445977	0.2574051	0.11355136	0.44536431	0.01706027	0.10407279	0.27863308	0.22551929	0.21801316	0.21558635	0.62474766	0.15360729	0.19496112	0.31916785	0.5683815	0.54751587	0.40831448	0.75558775	0.3102529	0.65344187	0.36016688	0.24573215	0.67354333	0.97627069	0.33825406	0.47272192	0.22258538	0.09470236	0.21035155	0.01139348	0	0.61718851	0.54183676	0.25730203	0.3679874	0.11948613	0.25164039	0.03558013	0.10677168	0.10450559	0.08006427	0.02999051	0.2688399	0.04607853	0.16212678	0.02237619	0.24673987	0.1192577	0.0559585	0.30429665	0.73797162	0.13676627	0.28540655	0.03844101	0.2511136	0.64272745	0.02084274	0.25974667	0.04143247	0.95813108	0.1731634	0.05316931	0.06786448	0.27698364
C26	0.07694858	0.1601194	0.22054913	0.29397835	0.07398508	0.29648785	0.49599191	0.35653497	0.32362449	0.01297044	0.07923281	0.32403612	0.0204495	0.23186683	0.34274571	0.1658189	0.01367953	0.23781828	0.53215852	0.26627429	0.81531983	0.28097685	0.1627239	0.38734514	0.48870156	0.5522916	0.62462252	0.25116042	0.41707196	0.19411362	0.35307838	0.62991016	0.61718851	0	0.2558863	0.79163062	0.16448942	0.07898222	0.64735675	0.50445595	0.33070405	0.40016257	0.28789651	0.8894181	0.04442546	0.325772	0.22419168	0.20417945	0.24694662	0.19302279	0.13890103	0.4269585	0.25993911	0.12998049	0.412407	0.48059887	0.83389613	0.16741487	0.42249736	0.33857914	0.41779137	0.14760665	0.97040268	0.14288901	0.2437978	0.33071565
C16.1.OH	0.14813583	0.49491245	0.0217101	0.08295975	0.03003122	0.25792308	0.11616502	0.049363	0.13641664	0.02927211	0.62039553	0.25280418	0.60938349	0.45106903	0.02903761	0.78497208	0.38996752	0.57666643	0.8753286	0.68217776	0.24841533	0.32786412	0.57059739	0.0093304	0.09248546	0.44272309	0.14613087	0.02548493	0.01064542	0.39381823	0.77214825	0.17214077	0.54183676	0.2558863	0	0.58442852	0.21825754	0.03721115	0.20060622	0.50685485	0.13682746	0.15127086	0.03990767	0.44981661	0.18000354	0.18386513	0.11720034	0.71541103	0.62245847	0.19167628	0.15520732	0.87885856	0.78952043	0.10940516	0.31644079	0.61065365	0.53993136	0.47715307	0.2325344	0.10900211	0.18746547	0.48627202	0.15455282	0.2911855	0.0884787	0.12209968
His	0.3044568	0.22446991	0.26777797	0.16673858	0.10128887	0.10495215	0.05649937	0.01968158	0.29728003	0.23052477	0.62999884	0.02972909	0.51566371	0.4745207	0.02751126	0.55940719	0.8547169	0.77254648	0.40312225	0.53707149	0.12616793	0.68668144	0.45745241	0.13627402	0.60406634	0.11719226	0.02169929	0.12369462	0.40365547	0.18690344	0.0981854	0.14630216	0.25730203	0.79163062	0.58442852	0	0.40990124	0.18216613	0.68406631	0.42716284	0.30171476	0.47013263	0.01959341	0.0905152	0.54397395	0.6443689	0.60224996	0.2148711	0.15861794	0.83703911	0.42547999	0.20459613	0.33300493	0.28805376	0.82020109	0.15233485	0.81143569	0.25075144	0.27812334	0.47693687	0.34187191	0.02832784	0.37619786	0.94579775	0.71977399	0.387533
Eubacterium	0.18228263	0.19467901	0.09294894	0.11083471	0.20193477	0.3023552	0.26012715	0.36972087	0.46421259	0.0861879	0.1064338	0.37115412	0.20313647	0.38521924	0.60017054	0.29980365	0.11114632	0.29924336	0.41160174	0.52982563	0.27800801	0.40633116	0.3484594	0.7873926	0.28904888	0.73332684	0.96379804	0.66213136	0.52536115	0.28760765	0.84307761	0.78737902	0.3679874	0.16448942	0.21825754	0.40990124	0	0.06810224	0.02973941	0.39043902	0.04683364	0.02520515	0.58428967	0.59104746	0.01442341	0.0121007	0.00525356	0.57196959	0.92267329	0.00413416	0.01511262	0.73691392	0.42420692	0.10251098	0.01208392	0.68700479	0.11461009	0.43459398	0.06060861	0.11129181	0.10802359	0.92129851	0.14085584	0.02441395	0.01513848	0.0363064
Acetobacter	0.00603008	0.01601961	0.00169369	0.00013693	0.0011794	0.00426233	0.00486661	0.00154335	0.04414031	0.00036729	0.04562136	0.01222061	0.04447918	0.07304068	0.01908512	0.08212906	0.07057845	0.14302301	0.14347243	0.15741592	0.02304042	0.18464562	0.19602783	0.06335101	0.11467359	0.07494645	0.07335639	0.07956618	0.11611468	0.03024632	0.23460901	0.18634562	0.11948613	0.07898222	0.03721115	0.18216613	0.06810224	0	0.01217346	0.19833604	0.00047514	0.00217461	0.00387034	0.15722331	0.00568539	0.00591534	0.00126529	0.12430574	0.31097613	0.0227473	0.00024633	0.20241863	0.05984436	0.00104422	0.07044723	0.16680798	0.21618623	0.15769554	0.00260624	0.0043893	0.00328479	0.07860505	0.04540976	0.04370706	0.00519563	0.00128246
Acidovorax	0.07236056	0.05136425	0.01712172	0.00261836	0.17153394	0.03130381	0.03704658	0.06571969	0.11050486	0.12902557	0.11881721	0.10848521	0.18048882	0.13267977	0.3262292	0.11023391	0.34128039	0.14253958	0.10518205	0.17057891	0.03765915	0.35487346	0.4660525	0.32403977	0.232355	0.17136869	0.19177865	0.63944071	0.38156598	0.09282275	0.49264479	0.51746194	0.25164039	0.64735675	0.20060622	0.68406631	0.02973941	0.01217346	0	0.062216	0.00052259	0.00070864	0.17485074	0.24650579	0.04286383	0.00702176	0.00053537	0.35785983	0.09301441	0.02679227	0.00159196	0.03257871	0.10579346	0.01279731	0.00398561	0.03841872	0.03998084	0.55316387	0.00133283	0.0012613	0.00268677	0.51347763	0.00386894	0.10943426	0.0195453	0.0004307
Cetobacteriu	0.2145281	0.1944627	0.11449922	0.1082537	0.23522089	0.18241785	0.02922819	0.13025619	0.17391512	0.59574501	0.29199573	0.31235494	0.85662187	0.20979882	0.21458749	0.37313932	0.6957424	0.38899619	0.16165056	0.57207959	0.04736366	0.32452028	0.58285689	0.34999681	0.16103694	0.38675242	0.48266335	0.46491182	0.23129672	0.64040136	0.53556023	0.13636896	0.03558013	0.50445595	0.50685485	0.42716284	0.39043902	0.19833604	0.062216	0	0.03624237	0.01905636	0.13546136	0.01194854	0.49244935	0.01686896	0.10192273	0.63656575	0.00268118	0.13432088	0.11249079	0.00957182	0.46054913	0.13284109	0.40741626	0.00181185	0.01076847	0.78234513	0.02108228	0.12046991	0.02569993	0.94837649	0.03265882	0.18472908	0.04343459	0.09208383
Dechloromo	0.00604858	0.00522169	0.00201351	5.49E-05	0.015375	0.00205627	0.00303751	0.00797744	0.02666113	0.02582235	0.01913258	0.02222954	0.05419754	0.04401216	0.06591248	0.04027703	0.08801561	0.05875161	0.02907126	0.0843253	0.00723207	0.10290202	0.15083375	0.15005026	0.05919675	0.05377185	0.12919124	0.19226392	0.16184742	0.08860768	0.23199229	0.23174529	0.10677168	0.33070405	0.13682746	0.30171476	0.04683364	0.00047514	0.00052259	0.03624237	0	1.23E-06	0.02848167	0.06882454	0.00990761	0.00037924	4.94E-05	0.2636831	0.07294756	0.01952174	9.57E-05	0.03614392	0.03983705	0.00035827	0.04441973	0.03764306	0.05684359	0.17893174	5.12E-05	0.00119427	0.00061416	0.19540508	0.00670823	0.06121462	0.00367089	8.63E-06
Dechlorosor	0.01135974	0.01296786	0.00491966	0.00049503	0.02253967	0.00579723	0.00681335	0.02041636	0.04172536	0.04331248	0.02036468	0.04978608	0.08692968	0.05918912	0.09044683	0.05801571	0.081764	0.07072135	0.03346426	0.12688174	0.00755233	0.09348547	0.17886517	0.22027331	0.04197827	0.07937913	0.26640148	0.21663483	0.17384437	0.17625272	0.35187214	0.2724473	0.10450559	0.40016257	0.15127086	0.47013263	0.02520515	0.00217461	0.00070864	0.01905636	1.23E-06	0	0.04941012	0.06537632	0.015465	2.36E-05	5.63E-05	0.38793572	0.07333957	0.00859085	0.00034114	0.03955385	0.05878593	0.00148873	0.05416146	0.04625969	0.021477	0.22112241	0.00018236	0.00307535	0.00100012	0.33455609	0.00874903	0.03847369	0.00104097	5.83E-05
Dorea	0.03218849	0.10813058	0.02076415	0.01144817	0.00126235	0.02238056	0.00652154	0.00083548	0.05298334	0.01234771	0.23384906	0.03782222	0.28056681	0.12745162	0.00101388	0.27050537	0.2603783	0.38302549	0.32825042	0.37167444	0.03783264	0.25185018	0.41065376	0.01247808	0.1309382	0.08857959	0.04521053	0.01853874	0.04729999	0.14681864	0.19963415	0.05534216	0.08006427	0.28789651	0.03990767	0.01959341	0.58428967	0.00387034	0.17485074	0.13546136	0.02848167	0.04941012	0	0.07890551	0.18010191	0.06412692	0.09997064	0.17667229	0.18186145	0.1628543	0.04941551	0.18884538	0.17667542	0.02646317	0.57199253	0.11826551	0.33562386	0.30743251	0.04367963	0.05369196	0.02187088	0.06712728	0.15934458	0.14772416	0.03734241	0.06669366
Dubosiella	0.21106658	0.09985848	0.05534123	0.12249884	0.05378529	0.16490401	0.00206938	0.01964693	0.23947746	0.27518124	0.28917492	0.02622528	0.69994032	0.44040207	0.02349776	0.60082844	0.6011833	0.55394651	0.25800232	0.70492972	0.02481201	0.31788949	0.11311806	0.22789503	0.13093734	0.34420781	0.20777245	0.08535566	0.15119912	0.37991888	0.52689444	0.02649046	0.02999051	0.8894181	0.44981661	0.0905152	0.59104746	0.15722331	0.24650579	0.01194854	0.06882454	0.06537632	0.07890551	0	0.18326214	0.06890614	0.12057215	0.49756815	0.01456214	0.50327016	0.14753629	0.11892112	0.71341596	0.14819545	0.63342226	0.02464374	0.38223499	0.10162398	0.03526016	0.41118377	0.18079661	0.60467377	0.0234028	0.5446011	0.18349961	0.10835595
Flavobacteri	0.03336981	0.01118096	0.00922411	0.01585619	0.01667149	0.04428537	0.02961019	0.0345364	0.18118336	0.00449058	0.02515006	0.00942208	0.02319303	0.23868346	0.10910678	0.1396243	0.04065935	0.1451552	0.16116106	0.19907132	0.07820911	0.19469985	0.02417937	0.3367245	0.15306916	0.22404189	0.24986716	0.15637698	0.30033605	0.04923464	0.49350443	0.35213133	0.2688399	0.04442546	0.18000354	0.54397395	0.01442341	0.00568539	0.04286383	0.49244935	0.00990761	0.015465	0.18010191	0.18326214	0	0.02309632	0.00098496	0.25889641	0.62208272	0.09158357	0.0028454	0.5858001	0.19226151	0.01295981	0.04037607	0.48559587	0.56619713	0.0157255	0.01507674	0.09008794	0.10698113	0.21207019	0.05075574	0.16686819	0.06169557	0.00424699
Hydrogenop	0.01847368	0.0263265	0.01327023	0.00699362	0.02224657	0.02959399	0.01412032	0.04779683	0.08280452	0.04548089	0.01883297	0.10796232	0.12890559	0.06986394	0.11825724	0.0819498	0.06241395	0.11353361	0.06304701	0.24038673	0.02400196	0.11658387	0.20452518	0.30384628	0.06117699	0.20375811	0.51347671	0.24231781	0.18517844	0.23510112	0.42099951	0.23572728	0.04607853	0.325772	0.18386513	0.6443689	0.0121007	0.00591534	0.00702176	0.01686896	0.00037924	2.36E-05	0.06412692	0.06890614	0.02309632	0	0.00059885	0.3440394	0.15964304	0.00127163	0.00079766	0.10271027	0.11481887	0.00628397	0.07993251	0.08664859	0.01171933	0.28647211	0.0008721	0.01540017	0.00200312	0.5422399	0.03524742	0.00671108	5.40E-05	0.00285476
Hyphomicro	0.01917234	0.01212211	0.00245122	0.00095892	0.0248529	0.01520635	0.01038379	0.0221554	0.08289384	0.01734561	0.02669642	0.02739579	0.06097111	0.11667127	0.11840452	0.0875481	0.08192978	0.09703606	0.08327805	0.15719011	0.02006229	0.16159423	0.12276162	0.25466864	0.0860173	0.1451031	0.22974406	0.24230923	0.21956313	0.07630742	0.48676286	0.32692938	0.16212678	0.22419168	0.11720034	0.60224996	0.00525356	0.00126529	0.00053537	0.10192273	4.94E-05	5.63E-05	0.09997064	0.12057215	0.00098496	0.00059885	0	0.31397781	0.2045623	0.01328589	5.30E-05	0.14560943	0.10725651	0.00238875	0.011047	0.12620475	0.09738118	0.13803168	0.00042243	0.00685779	0.0071188	0.3488479	0.00602206	0.05807964	0.00505966	1.77E-05
Lactobacillu	0.20138784	0.09957668	0.18630113	0.34782347	0.28564497	0.52422707	0.13586852	0.20964874	0.38836485	0.16909124	0.29830581	0.25095979	0.29246856	0.1511684	0.51562701	0.19348875	0.64604665	0.5361876	0.56182856	0.49691983	0.81272917	0.91197995	0.52418436	0.29809253	0.57219942	0.91252397	0.13546905	0.92918475	0.58577521	0.00168437	0.05021048	0.12035938	0.02237619	0.20417945	0.71541103	0.2148711	0.57196959	0.12430574	0.35785983	0.63656575	0.2636831	0.38793572	0.17667229	0.49756815	0.25889641	0.3440394	0.31397781	0	0.90959647	0.30703659	0.0785369	0.67694283	0.52070374	0.18286328	0.14757102	0.24242789	0.92860836	0.81058102	0.09541686	0.23592744	0.10385715	0.47310136	0.49354615	0.15612753	0.35285748	0.44543621
Lactococcus	0.33088173	0.22521649	0.15723382	0.10141954	0.36846491	0.11722372	0.03906308	0.09469283	0.16848325	0.81309858	0.51717495	0.16969872	0.88944534	0.36521669	0.18662914	0.49249383	0.96056011	0.42240792	0.15240635	0.42870352	0.0295174	0.37020058	0.54550572	0.29889565	0.17663948	0.15379524	0.16256274	0.42125578	0.31280932	0.6645555	0.49178014	0.22837525	0.24673987	0.24694662	0.62245847	0.15861794	0.92267329	0.31097613	0.09301441	0.00268118	0.07294756	0.07333957	0.18186145	0.01456214	0.62208272	0.15964304	0.2045623	0.90959647	0	0.66064385	0.276685	0.00139479	0.42326682	0.19418156	0.59670952	0.00092175	0.14420171	0.5755273	0.06672366	0.17143277	0.11465176	0.63752036	0.0162688	0.87139831	0.32155949	0.09776637
Megasphaer	0.07984431	0.17306889	0.09902155	0.05148182	0.10417424	0.14891761	0.17130117	0.23775631	0.25493982	0.06428326	0.07690445	0.45555311	0.20706139	0.12594581	0.40193131	0.14095854	0.09374815	0.25606578	0.25297765	0.44246604	0.18640618	0.34695357	0.67177778	0.52063419	0.27863226	0.49195152	0.93137644	0.58842807	0.39823778	0.28519548	0.61427632	0.57016305	0.1192577	0.19302279	0.19167628	0.83703911	0.00413416	0.0227473	0.02679227	0.13432088	0.01952174	0.00859085	0.1628543	0.50327016	0.09158357	0.00127163	0.01328589	0.30703659	0.66064385	0	0.00679195	0.34013254	0.1633317	0.05124068	0.06336059	0.35272156	0.00908856	0.82941017	0.02840269	0.02815794	0.00674763	0.80452164	0.246702	3.11E-05	6.65E-05	0.04512865
Methylovers	0.00892593	0.00479996	0.00269123	0.00159515	0.02024792	0.01876835	0.00712215	0.01848527	0.06532091	0.01010865	0.01781087	0.03280241	0.04143112	0.04752283	0.12965087	0.03940905	0.08328045	0.0910394	0.07741988	0.13386026	0.04865897	0.22578708	0.16128915	0.19633942	0.20729746	0.20365195	0.16360775	0.31925143	0.22835003	0.01851076	0.21404285	0.2164311	0.0559585	0.13890103	0.15520732	0.42547999	0.01511262	0.00024633	0.00159196	0.11249079	9.57E-05	0.00034114	0.04941551	0.14753629	0.0028454	0.00079766	5.30E-05	0.0785369	0.276685	0.00679195	0	0.14938266	0.07747433	0.0010342	0.00950312	0.09176108	0.11243096	0.23083742	5.93E-05	0.00317562	0.00089331	0.27936865	0.02219368	0.01852939	0.00341288	0.00068519
MND1	0.27566066	0.17756227	0.27024557	0.04415948	0.44915056	0.03781262	0.09682312	0.14588176	0.20858715	0.70723332	0.3358482	0.21986031	0.5282537	0.23037351	0.38723205	0.21029844	0.81725504	0.31462597	0.04142963	0.26170612	0.02414212	0.46469351	0.84542445	0.58629415	0.34573394	0.06045455	0.27709706	0.81182189	0.74314619	0.49184492	0.23420174	0.63737251	0.30429665	0.4269585	0.87885856	0.20459613	0.73691392	0.20241863	0.03257871	0.00957182	0.03614392	0.03955385	0.18884538	0.11892112	0.5858001	0.10271027	0.14560943	0.67694283	0.00139479	0.34013254	0.14938266	0	0.07807617	0.14729312	0.41329181	0.00272903	0.04102156	0.8440485	0.04493912	0.06101009	0.03146777	0.39691151	0.06221912	0.49952883	0.19182466	0.05947802
Negativibac	0.04081232	0.052401	0.31035211	0.02349693	0.19605536	0.00194759	0.32282409	0.23348928	0.10796966	0.18063284	0.02447718	0.22284613	0.00684523	0.05590299	0.42637837	0.00808415	0.04365069	0.04277392	0.00321836	0.01809555	0.10812021	0.12066827	0.42433484	0.66642353	0.24015424	0.00417426	0.47278225	0.59046012	0.8695373	0.40258025	0.04484411	0.83604311	0.73797162	0.25993911	0.78952043	0.33300493	0.42420692	0.05984436	0.10579346	0.46054913	0.03983705	0.05878593	0.17667542	0.71341596	0.19226151	0.11481887	0.10725651	0.52070374	0.42326682	0.1633317	0.07747433	0.07807617	0	0.03779854	0.48115836	0.40438821	0.19559986	0.37309612	0.12551531	0.03162375	0.05222062	0.01698634	0.52109238	0.22593103	0.14020391	0.04657955
Persicirhabd	1.24E-05	0.00047177	0.00061906	0.00226094	0.01190541	0.00547083	0.00930765	0.0204967	0.00087974	0.02452589	0.00291404	0.05305911	0.00950351	0.00289642	0.07333708	0.00513679	0.02228206	0.00525387	0.02281396	0.01032674	0.08001675	0.01936143	0.04738771	0.04262579	0.03940483	0.07559445	0.07140571	0.13000776	0.0490302	0.09556153	0.0836915	0.12321916	0.13676627	0.12998049	0.10940516	0.28805376	0.10251098	0.00104422	0.01279731	0.13284109	0.00035827	0.00148873	0.02646317	0.14819545	0.01295981	0.00628397	0.00238875	0.18286328	0.19418156	0.05124068	0.0010342	0.14729312	0.03779854	0	0.09158273	0.10204147	0.20047239	0.12863857	0.00175369	0.00078666	0.0028129	0.11160909	0.04316991	0.08736313	0.01823405	0.00141852
Prevotella_9	0.23831346	0.12785056	0.05713795	0.09527641	0.43957463	0.35114747	0.1634011	0.27134454	0.38929171	0.18442973	0.26302911	0.26159238	0.28199104	0.30715136	0.79613885	0.25173121	0.56278133	0.334337	0.47344372	0.4000652	0.39503437	0.86050424	0.53107959	0.53971353	0.83140357	0.86530241	0.30143773	0.86813922	0.62484397	0.02202149	0.7254722	0.58374485	0.28540655	0.412407	0.31644079	0.82020109	0.01208392	0.07044723	0.00398561	0.40741626	0.04441973	0.05416146	0.57199253	0.63342226	0.04037607	0.07993251	0.011047	0.14757102	0.59670952	0.06336059	0.00950312	0.41329181	0.48115836	0.09158273	0	0.22889188	0.29295793	0.75306626	0.02232989	0.03896825	0.05571583	0.99092596	0.03697301	0.14247783	0.12156067	0.03669281
Reyranella	0.20603347	0.10407837	0.07997896	0.08582551	0.33492921	0.16063881	0.01467238	0.07590351	0.12807609	0.61575069	0.37538516	0.172094	0.73516397	0.15514932	0.26392551	0.27167781	0.97672964	0.35642409	0.15941825	0.37561147	0.08550422	0.55023786	0.58593429	0.21497563	0.45062338	0.3714714	0.08891883	0.67391114	0.29624985	0.19932298	0.21790178	0.09368735	0.03844101	0.48059887	0.61065365	0.15233485	0.68700479	0.16680798	0.03841872	0.00181185	0.03764306	0.04625969	0.11826551	0.02464374	0.48559587	0.08664859	0.12620475	0.24242789	0.00092175	0.35272156	0.09176108	0.00272903	0.40438821	0.10204147	0.22889188	0	0.11421911	0.82528432	0.01107461	0.06768391	0.01950923	0.73505557	0.01366501	0.42068079	0.18414683	0.08811177
Ruminiclost	0.29003828	0.43144507	0.34688334	0.1231689	0.43800089	0.19544249	0.30882065	0.46821463	0.34529006	0.58161184	0.26147293	0.81245153	0.65823757	0.23696017	0.65263362	0.28232761	0.41857735	0.36785099	0.17364625	0.5573911	0.12324603	0.3919767	0.86379234	0.8301106	0.24168542	0.36583748	0.81299777	0.91902442	0.62660821	0.93108588	0.78076045	0.84107377	0.2511136	0.83389613	0.53993136	0.81143569	0.11461009	0.21618623	0.03998084	0.01076847	0.05684359	0.021477	0.33562386	0.38223499	0.56619713	0.01171933	0.09738118	0.92860836	0.14420171	0.00908856	0.11243096	0.04102156	0.19559986	0.20047239	0.29295793	0.11421911	0	0.70713584	0.08518032	0.06890516	0.02082116	0.85814588	0.2271902	0.0283937	0.00837703	0.10889794
Ruminococ	0.12344562	0.05532115	0.11131207	0.16310886	0.03950653	0.09675477	0.10257662	0.08738353	0.26848913	0.08373674	0.08749186	0.01307817	0.07352393	0.5275436	0.06012382	0.36721304	0.07117802	0.23700682	0.19200691	0.25867076	0.12985744	0.09668961	0.00148168	0.41913113	0.07522445	0.13889007	0.32024725	0.03279713	0.31799577	0.43474943	0.5285433	0.35827137	0.64272745	0.16741487	0.47715307	0.25075144	0.43459398	0.15769554	0.55316387	0.78234513	0.17893174	0.22112241	0.30743251	0.10162398	0.0157255	0.28647211	0.13803168	0.81058102	0.5755273	0.82941017	0.23083742	0.8440485	0.37309612	0.12863857	0.75306626	0.82528432	0.70713584	0	0.24781345	0.6144563	0.69300309	0.0992591	0.24095124	0.95047446	0.52201736	0.11341587
Simplicispira	0.01421485	0.00335623	0.00193833	0.00271627	0.03312172	0.02241629	0.00081648	0.01157363	0.04768495	0.05080176	0.03080007	0.02539547	0.11036291	0.04554608	0.10627107	0.05456086	0.19481808	0.09996114	0.05422765	0.14845663	0.02677426	0.22043796	0.14674174	0.16241104	0.1818868	0.2074632	0.10542295	0.32322573	0.18016808	0.03133408	0.18143039	0.10389328	0.02084274	0.42249736	0.2325344	0.27812334	0.06060861	0.00260624	0.00133283	0.02108228	5.12E-05	0.00018236	0.04367963	0.03526016	0.01507674	0.0008721	0.00042243	0.09541686	0.06672366	0.02840269	5.93E-05	0.04493912	0.12551531	0.00175369	0.02232989	0.01107461	0.08518032	0.24781345	0	0.00538516	0.00058621	0.37569799	0.00391026	0.05537474	0.00896527	0.00100058
Terrimonas	0.00564642	0.02408053	0.00989269	0.00260321	0.09539418	0.01381969	0.05139947	0.06434443	0.00755209	0.08622859	0.04696003	0.19266319	0.05920733	0.00935242	0.23664508	0.01562349	0.13896282	0.02629612	0.06209142	0.02800128	0.11291931	0.11847925	0.40200747	0.08133661	0.14161418	0.09808053	0.09560293	0.42875928	0.14218244	0.11523475	0.15633936	0.34500186	0.25974667	0.33857914	0.10900211	0.47693687	0.11129181	0.0043893	0.0012613	0.12046991	0.00119427	0.00307535	0.05369196	0.41118377	0.09008794	0.01540017	0.00685779	0.23592744	0.17143277	0.02815794	0.00317562	0.06101009	0.03162375	0.00078666	0.03896825	0.06768391	0.06890516	0.6144563	0.00538516	0	0.00029643	0.21991083	0.05058523	0.07227821	0.01771348	0.00326878
Thauera	0.01175824	0.02378284	0.01449566	0.00397823	0.05923211	0.02198806	0.01661828	0.03875585	0.0296392	0.0751697	0.04892686	0.15451831	0.12907425	0.01412051	0.17418498	0.02986214	0.18879323	0.08936224	0.06464991	0.11995742	0.06553246	0.22291137	0.47574467	0.13100985	0.22250352	0.17337424	0.16543404	0.44460177	0.1865869	0.08163884	0.12188822	0.19651774	0.04143247	0.41779137	0.18746547	0.34187191	0.10802359	0.00328479	0.00268677	0.02569993	0.00061416	0.00100012	0.02187088	0.18079661	0.10698113	0.00200312	0.0071188	0.10385715	0.11465176	0.00674763	0.00089331	0.03146777	0.05222062	0.0028129	0.05571583	0.01950923	0.02082116	0.69300309	0.00058621	0.00029643	0	0.3382855	0.05074625	0.01306528	0.0023268	0.00756003
Tyzzerella_4	0.09464285	0.12020964	0.29110698	0.06248793	0.07629945	0.00953066	0.2568129	0.07534258	0.16308885	0.0752232	0.1818963	0.05441796	0.03037299	0.25534965	0.09247892	0.14300921	0.13547789	0.22600296	0.12446896	0.08929815	0.15439839	0.22448596	0.27518742	0.27043913	0.29699766	0.00461453	0.10734508	0.12822579	0.5808708	0.28959492	0.0733428	0.77951523	0.95813108	0.14760665	0.48627202	0.02832784	0.92129851	0.07860505	0.51347763	0.94837649	0.19540508	0.33455609	0.06712728	0.60467377	0.21207019	0.5422399	0.3488479	0.47310136	0.63752036	0.80452164	0.27936865	0.39691151	0.01698634	0.11160909	0.99092596	0.73505557	0.85814588	0.0992591	0.37569799	0.21991083	0.3382855	0	0.67423191	0.83788054	0.56032319	0.171752
uncultured_	0.15595886	0.07296765	0.00546505	0.01651747	0.12415343	0.09362744	0.00378645	0.01824479	0.14450415	0.18945248	0.29949797	0.02719607	0.49495006	0.35583414	0.11081628	0.42476786	0.67502385	0.33231022	0.27629263	0.40194935	0.02208792	0.39968623	0.22416652	0.16717551	0.15810288	0.2878062	0.06732214	0.28341557	0.1663266	0.12425769	0.77655746	0.14826991	0.1731634	0.97040268	0.15455282	0.37619786	0.14085584	0.04540976	0.00386894	0.03265882	0.00670823	0.00874903	0.15934458	0.0234028	0.05075574	0.03524742	0.00602206	0.49354615	0.0162688	0.246702	0.02219368	0.06221912	0.52109238	0.04316991	0.03697301	0.01366501	0.2271902	0.24095124	0.00391026	0.05058523	0.05074625	0.67423191	0	0.47434521	0.110526	0.00424423
uncultured_	0.09342012	0.19871617	0.16331222	0.13181208	0.11553947	0.26102194	0.21404066	0.30781295	0.30643112	0.07938893	0.08767015	0.57691048	0.24346184	0.10654879	0.44276412	0.14440919	0.10544732	0.32576585	0.32653101	0.53595622	0.35013201	0.43473932	0.71731462	0.52333109	0.45626287	0.71104653	0.99551926	0.64155502	0.41924006	0.26565682	0.43766815	0.43633977	0.05316931	0.14288901	0.2911855	0.94579775	0.02441395	0.04370706	0.10943426	0.18472908	0.06121462	0.03847369	0.14772416	0.5446011	0.16686819	0.00671108	0.05807964	0.15612753	0.87139831	3.11E-05	0.01852939	0.49952883	0.22593103	0.08736313	0.14247783	0.42068079	0.0283937	0.95047446	0.05537474	0.07227821	0.01306528	0.83788054	0.47434521	0	0.00077034	0.14730089
uncultured_	0.0344777	0.10395887	0.03015887	0.01215321	0.01971714	0.05211235	0.04179936	0.0631989	0.12531159	0.02760818	0.06140057	0.20956728	0.20660254	0.10220982	0.11220626	0.14996056	0.07859457	0.22315701	0.18460513	0.39238664	0.04801404	0.19396492	0.46280477	0.25894354	0.09309736	0.25638507	0.61655924	0.22367507	0.17468272	0.28769341	0.55123754	0.29908426	0.06786448	0.2437978	0.0884787	0.71977399	0.01513848	0.00519563	0.0195453	0.04343459	0.00367089	0.00104097	0.03734241	0.18349961	0.06169557	5.40E-05	0.00505966	0.35285748	0.32155949	6.65E-05	0.00341288	0.19182466	0.14020391	0.01823405	0.12156067	0.18414683	0.00837703	0.52201736	0.00896527	0.01771348	0.0023268	0.56032319	0.110526	0.00077034	0	0.01434375
uncultured_	0.01529019	0.01103504	0.00328617	4.30E-05	0.02435948	0.00178609	0.0090642	0.0112197	0.04692759	0.02656484	0.03259028	0.01525762	0.04963421	0.10581306	0.07882998	0.07222923	0.09499292	0.07087578	0.04218239	0.08820506	0.0055457	0.11056109	0.13262589	0.20214574	0.04765019	0.03574954	0.13450071	0.1831885	0.21453251	0.11372869	0.38931703	0.38780541	0.27698364	0.33071565	0.12209968	0.387533	0.0363064	0.00128246	0.0004307	0.09208383	8.63E-06	5.83E-05	0.06669366	0.10835595	0.00424699	0.00285476	1.77E-05	0.44543621	0.09776637	0.04512865	0.00068519	0.05947802	0.04657955	0.00141852	0.03669281	0.08811177	0.10889794	0.11341587	0.00100058	0.00326878	0.00756003	0.171752	0.00424423	0.14730089	0.01434375	0

*: p≤0.05; **: p≤0.01; ***: p≤0.001
